# Signal Transduction at the Domain Interface of Prokaryotic Pentameric Ligand-Gated Ion Channels

**DOI:** 10.1371/journal.pbio.1002393

**Published:** 2016-03-04

**Authors:** Carlo Bertozzi, Iwan Zimmermann, Sibylle Engeler, Ricarda J. C. Hilf, Raimund Dutzler

**Affiliations:** Department of Biochemistry, University of Zürich, Zürich, Switzerland; Institut Pasteur, FRANCE

## Abstract

Pentameric ligand-gated ion channels are activated by the binding of agonists to a site distant from the ion conduction path. These membrane proteins consist of distinct ligand-binding and pore domains that interact via an extended interface. Here, we have investigated the role of residues at this interface for channel activation to define critical interactions that couple conformational changes between the two structural units. By characterizing point mutants of the prokaryotic channels ELIC and GLIC by electrophysiology, X-ray crystallography and isothermal titration calorimetry, we have identified conserved residues that, upon mutation, apparently prevent activation but not ligand binding. The positions of nonactivating mutants cluster at a loop within the extracellular domain connecting β-strands 6 and 7 and at a loop joining the pore-forming helix M2 with M3 where they contribute to a densely packed core of the protein. An ionic interaction in the extracellular domain between the turn connecting β-strands 1 and 2 and a residue at the end of β-strand 10 stabilizes a state of the receptor with high affinity for agonists, whereas contacts of this turn to a conserved proline residue in the M2-M3 loop appear to be less important than previously anticipated. When mapping residues with strong functional phenotype on different channel structures, mutual distances are closer in conducting than in nonconducting conformations, consistent with a potential role of contacts in the stabilization of the open state. Our study has revealed a pattern of interactions that are crucial for the relay of conformational changes from the extracellular domain to the pore region of prokaryotic pentameric ligand-gated ion channels. Due to the strong conservation of the interface, these results are relevant for the entire family.

## Abstract 2

Interacting residues at the interface between the extracellular domain and the pore of prokaryotic pentameric ligand-gated ion channels play an important role in the transduction of conformational changes that lead to channel activation.

## Author Summary

The pentameric ligand-gated ion channels constitute a large family of membrane proteins that are expressed in animals and certain bacteria. Their molecular architecture and function is conserved throughout the family. In mammals, they operate as receptors of the neurotransmitters acetylcholine, serotonin, GABA, and glycine and play a key role in electrical signal transduction at chemical synapses. These receptors are called ionotropic because they open a selective ion conduction path across the membrane upon binding of the neurotransmitters to a site that is exposed to the extracellular medium. Ligand binding promotes a conformational change in the extracellular domain that is transmitted over more than 50 Å to the pore domain. Due to this long-range effect, pentameric ligand-gated ion channels have become important model systems for the study of allosteric processes, a mechanism that is of large importance for biology and entails the regulation of a protein activity by an effector that binds to a distant domain. In the present study, we investigated the role that residues in the contact region between the ligand-binding and the pore domains of two bacterial pentameric ligand-gated ion channels of known structure have in the transduction of conformational changes. Our study shows that single mutations severely influence the functional properties, with certain mutations preventing activation. The results underline the importance of highly conserved residues in the domain interface for the transmission of allosteric signals and thus likely apply also to other family members.

## Introduction

During activation of a pentameric ligand-gated ion channel (pLGIC), the binding of agonists promotes the opening of a selective ion conduction pore at a distance of more than 50 Å away from the binding sites [1,2]. This process has been described by means of the Monod Weyman Changeux (MWC) mechanism of allosteric proteins, where activation can be broken down into distinct steps defining ligand binding and the shift in the equilibrium between the open and closed state of the pore [3–5]. pLGICs constitute a large family of membrane proteins that are expressed in animals and certain prokaryotes [6]. In mammals, the family encompasses ionotropic neurotransmitter receptors for acetylcholine, serotonin, GABA, and glycine, which are key players in electrical signal transduction at chemical synapses [7], whereas prokaryotic pLGICs are potentially involved in pH resistance [8,9]. All family members share a conserved molecular architecture composed of five either identical or closely related subunits. Over recent years, insight into the structural properties of pLGICs has been obtained from different sources. Electron microscopy studies of the nicotinic acetylcholine receptor (nAChR) from Torpedo electric ray have shed light on the structure of a heteropentameric receptor at medium resolution [10,11]. A recent study by single-particle electron cryomicroscopy revealed agonist and antagonist bound views of the glycine receptor (GlyR) [12]. Structures at higher resolution have been provided by X-ray crystallography for various pro- and eukaryotic family members [13–21]. Although these structures show different conformations of the channels, whose assignment to defined functional states is in certain cases still ambiguous [22], they closely resemble each other with respect to their general architecture. Each subunit consists of a predominantly β-stranded extracellular domain and an α-helical transmembrane pore, which interact via an extended interface. Both domains constitute independent folding units that, in certain cases, can be expressed as isolated proteins, thereby maintaining their respective structure observed in the full-length receptors [23–25]. The acetylcholine binding protein, which resembles the extracellular domain, even is an independent soluble protein [26]. Besides their close structural relationship, family members also share a common gating mechanism. Whereas the probability for channel opening in the ligand-free state is very low, it is increased by several orders of magnitude following agonist binding to sites in the extracellular domain located at the boundary between two adjacent subunits [1,5]. Since conformational rearrangements in this part of the protein are transduced via the domain interface to the transmembrane pore [27], it is not surprising that the residues at this interface belong to the most conserved parts of the protein.

In this study, we were interested in the role of interactions at the domain interface for the transduction of conformational changes in pLGICs. For that purpose, we have characterized mutants of ELIC and GLIC, two prokaryotic family members, by electrophysiology, calorimetry, and X-ray crystallography. These prokaryotic channels are ideal targets for mechanistic investigations: Their detailed structures have been determined in different conformations and show compact proteins that contain the main features of pLGICs [28]. Moreover, unlike many eukaryotic pLGICs, they form functional homopentamers that have been characterized on a macroscopic and a single channel level and that exhibit a functional behavior that closely resembles family members of higher organisms [8,9,29,30]. Whereas ELIC forms a cation-selective channel with high conductance that is activated with high efficacy by the primary amines cysteamine, propylamine, and GABA [9], the cation-selective GLIC is activated by protons and inhibited by bulky positively charged compounds that also act as open channel blockers of the nAChR [8,31]. Our study has identified a cluster of interacting residues located at the β1-β2 turn, the β6-β7 loop and the pre-M1 region of the extracellular domain, and the M2-M3 loop of the pore that exert a strong influence on channel function. These residues face a tightly packed core of the subunit, suggesting that their mutual interactions are critical for the transduction of signals underlying channel activation. Our results are generally consistent with previous investigations on eukaryotic receptors, which underlines the conservation of the activation mechanism throughout the family.

## Results

### Alanine Scanning Mutagenesis at the Domain Interface

**Fig 1:**
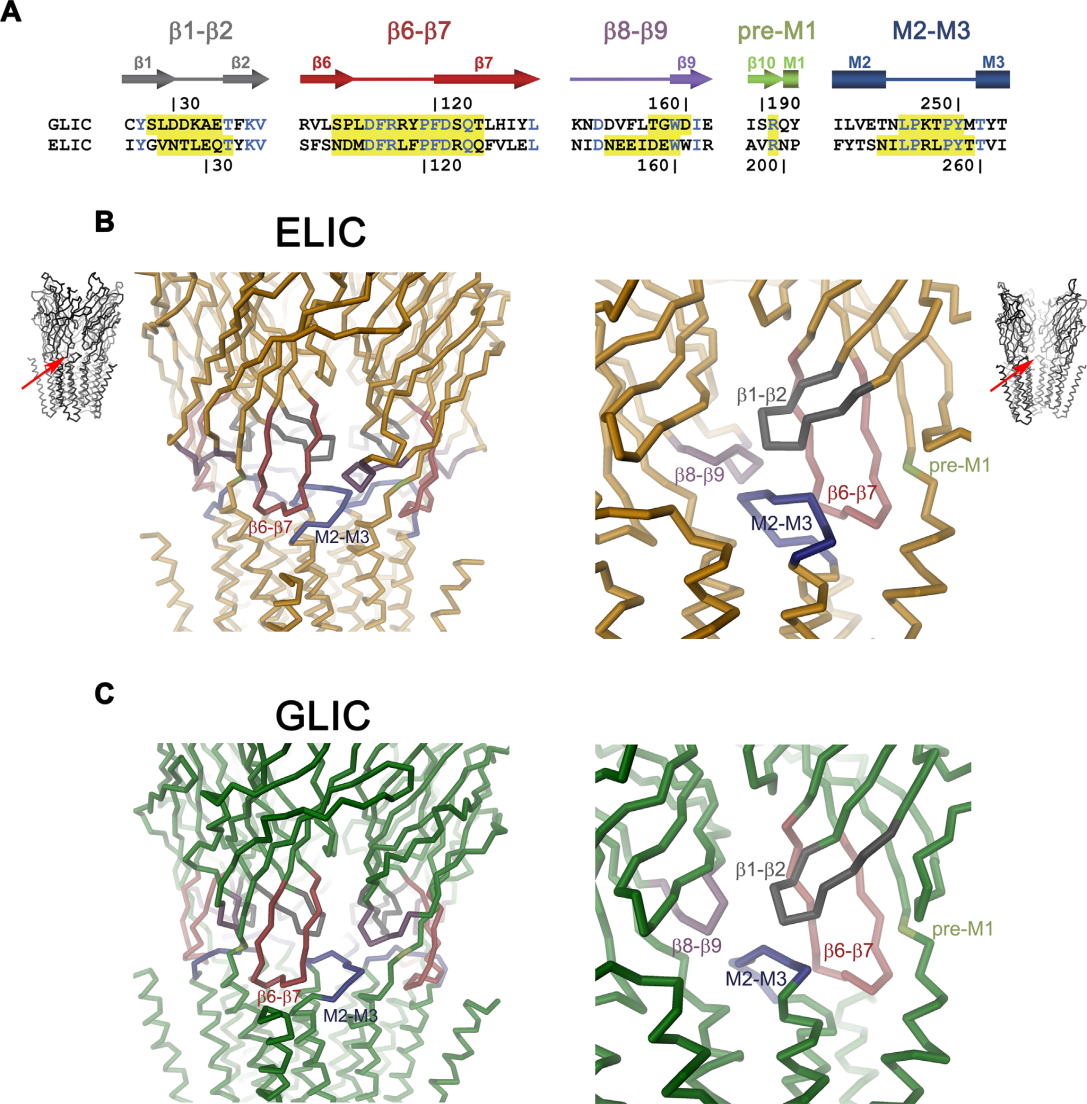
Domain Interface of pLGICs. (A) Aligned sequences of the domain interface of GLIC and ELIC with residue numbering shown above and below, respectively. Secondary structure elements are indicated. Identical residues are colored in blue. Residues mutated in this study in either protein are highlighted in yellow. (B) Domain interface of ELIC (Protein Data Bank entry 2VL0). The protein is displayed as Cα-trace, mutated regions are shown in unique colors, the views are indicated. Left: View at the interface from the outside. Right: View from the pore region on two adjacent subunits. (C) Domain interface of GLIC (Protein Data Bank entry 3EHZ). The protein is displayed as Cα-trace, with mutated regions shown in unique colors. The views are as in B.

**Fig 2:**
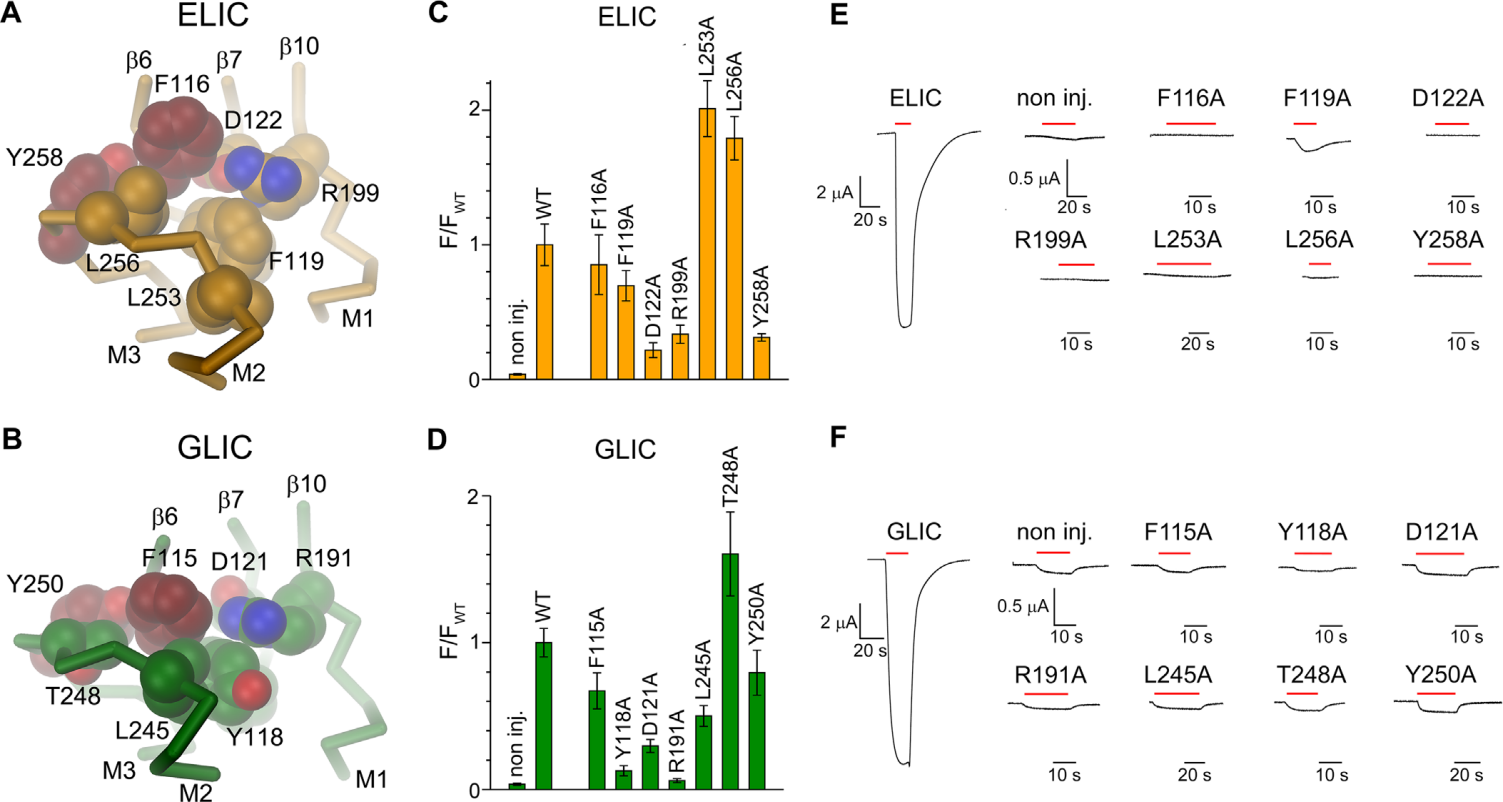
Nonactivating mutations at the domain interface. Cα-trace of parts of the domain interface in a single subunit of (A), ELIC and (B), GLIC with sidechains of selected nonactivating mutants shown as CPK models. The views are from within the pore (as in Fig 1B, right panel). Residues in ELIC for which alanine mutants were characterized in detail (Phe116 and Tyr258) and the corresponding residues in GLIC (Phe115 and Tyr250) are colored in red. (C–D), Surface expression of nonactivating mutants of (C), ELIC and (D), GLIC. Data show averages of 7–14 different oocytes and are normalized to wild type (WT). Background of noninjected (non inj.) oocytes was not subtracted. Errors are standard error of the mean (SEM). (E–F), Current response of a representative oocyte at high agonist concentration that was subsequently used for the analysis of surface expression. Currents were recorded at −40 mV. (E) Response in ELIC after application of 25 mM cysteamine. (F) Response in GLIC after change to pH 4. Application of agonist is indicated by a red bar. (See S1 Data for the raw data used to generate plots shown in panels C and D).

To investigate the role of interactions between the ligand-binding and the pore domain of pLGICs, we have selected residues in ELIC and GLIC that are either part of the domain interface or that are located in close proximity (Fig 1 and S1 Fig). In an initial screen, we have mutated these residues to alanine and expressed them in *Xenopus laevis* oocytes. Surface expression was quantified by ELISA with an antibody that recognizes a tag fused to the extracellular N-terminus of the respective protein. Most constructs showed robust expression, with few exceptions where the truncation of the side chain has led to a strong reduction of the ELISA signal (S2 Fig). To probe whether the mutated proteins would still be activated by ligands, we have measured the current response upon application of agonist by two-electrode voltage clamp electrophysiology. Whereas the majority of the investigated constructs showed response at high agonist concentration, the mutation of certain positions, although well expressed, resulted in either an apparent loss of activation or very low currents (Fig 2 and S2 Fig). In general, equivalent positions in ELIC and GLIC exhibited a similar pattern, which underlines the role of conserved residues at the domain interface for channel activation, but there were also some differences observed. In both cases, mutants with strongly compromised activation properties cluster at the loops connecting β-strands 6 and 7 (the cys-loop of eukaryotic receptors that contains the region of highest conservation) and α-helices M2 and M3 of the pore domain (the M2-M3 loop). A nonactivating phenotype was also found for certain residues of the β8-β9 loop that connect to the neighboring subunit and in GLIC, for an aspartate in the β1-β2 turn. Finally, no activation in case of ELIC and no expression in case of GLIC was observed for a strictly conserved arginine at the end of β-10 (the pre-M1 region). None of the investigated mutations showed detectable basal activity in the absence of agonists.

### Characterization of Nonactivating Mutants at the Domain Interface

**Fig 3:**
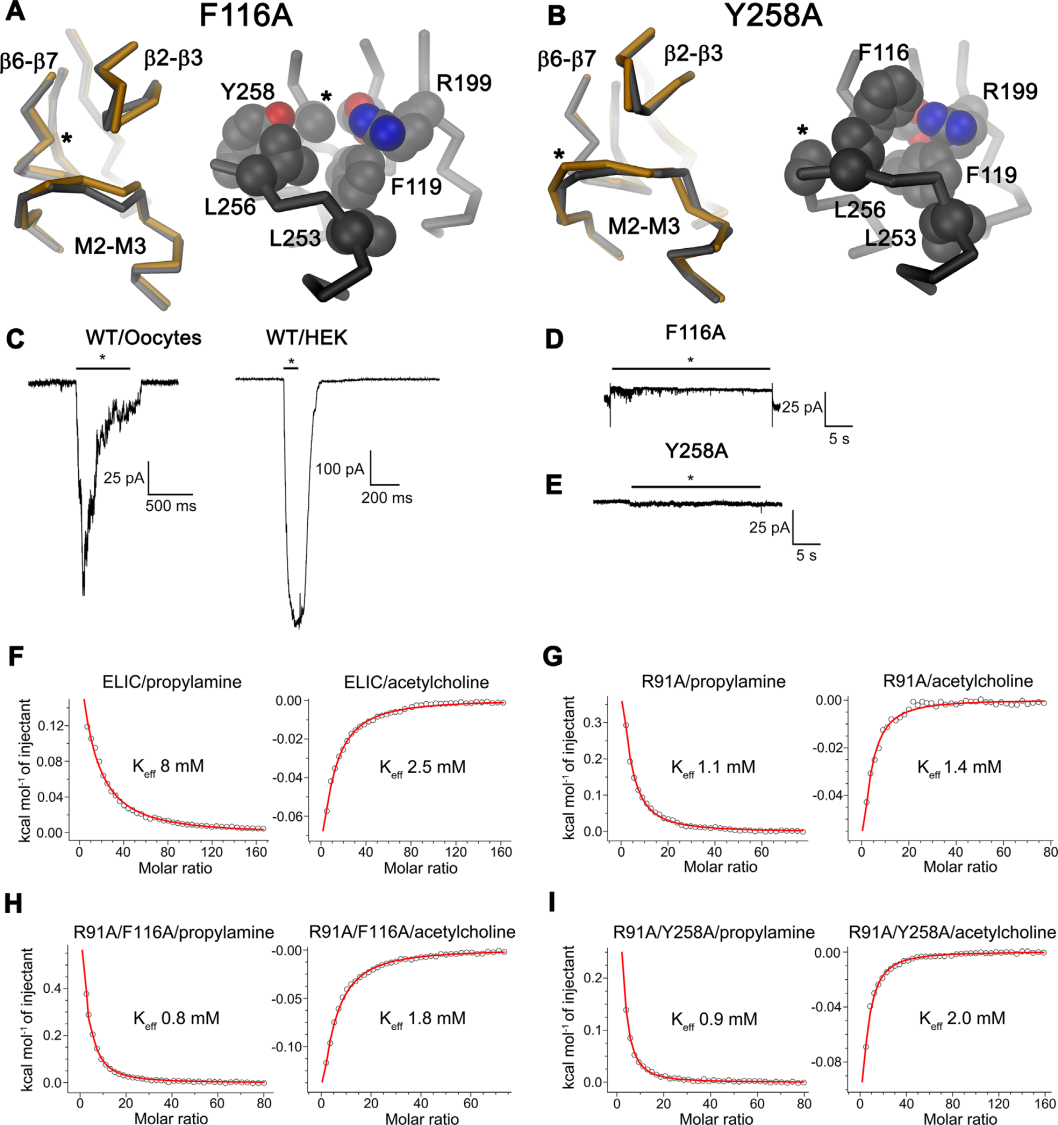
Characterization of two nonactivating mutants of ELIC. (A) Refined structure of the ELIC mutant F116A. Left, Cα-trace of part of a single subunit of F116A (grey) showing the domain interface superimposed on ELIC WT (orange). Right, interface of F116A with sidechains of selected residues shown as CPK models. View and selection are as in Fig 2A. (B) Analogous representation of the refined structure of the mutant Y258A. In A and B, the site of mutation is indicated by an asterisk. (C) Macroscopic currents recorded from representative patches of ELIC WT upon fast application and washout of agonist. Left, current response of a membrane patch of *X*. *laevis* oocytes expressing ELIC upon application of 20 mM cysteamine. Right, current recorded from a membrane patch of ELIC expressed in human embryonic kidney 293 (HEK293) cells upon application of 25 mM propylamine. The difference in magnitude of the currents is due to the higher expression of ELIC in HEK293 cells. (D) Representative current response of a membrane patch of the ELIC mutant F116A expressed in *X*. *laevis* oocytes upon application of 20 mM cysteamine. (E) Representative current response of a membrane patch of the ELIC mutant Y258A expressed in HEK293 cells upon application of 25 mM propylamine. C–E, Currents were recorded from excised patches in the outside-out configuration at a potential of −50 mV for HEK cell and −80 mV for *X*. *laevis* oocyte patches. Application of agonist by fast solution exchange is indicated by a black bar. (F–I), ITC experiments. Agonist and antagonist binding to (F), ELIC WT, (G), the ligand binding site mutant R91A, the double mutants (H), R91A/F116A and (I), R91A/Y258A. Graphs show a fit of the integrated and corrected heat upon addition of the agonist propylamine (left) and the antagonist acetylcholine (right) to a binding isotherm (red line). Experiments were repeated twice with similar results. (See S1 Data for the raw data used to generate plots shown in panels F–I).

When mapped on the structure, most mutations resulting in nonactivation by agonist point into a tightly packed core of the protein, irrespectively of their position in the sequence, thus suggesting that any disruption of this core may interfere with channel activation (Fig 2A and 2B and S3A Fig). To exclude that these mutations cause misfolding of the protein, we have expressed several of them in *Escherichia coli*. Most mutants showed wild type (WT)-like expression levels and were stable in detergent solution. For two cases, the ELIC mutants F116A of the β6-β7 loop and Y258A of the M2-M3 loop, we have grown crystals and determined structures at 3.5 and 3.2 Å, respectively (Table 1). Both mutants crystallized in the same nonconducting conformation that has been observed in all known ELIC structures. Small structural differences in the vicinity of the respective mutations indicate local rearrangements of protein interactions due to the loss of the bulky aromatic side chains (Fig 3A and 3B, S4A and S4B Fig). The data suggests that the mutations, despite their severe phenotype on channel activation, have only a local effect on the protein structure. We have also investigated whether both mutants would at least show residual activity, and we have thus expressed them in *X*. *laevis* oocytes and HEK-293 cells and studied excised patches in the outside-out configuration upon fast application of agonist. Neither of the two mutants display ligand-induced channel activity in any of numerous independent recordings (Fig 3C–3E). To exclude that the two nonactivating mutations have compromised the ability of the protein to recognize its ligand, we have studied agonist and antagonist binding to the detergent solubilized protein by isothermal titration calorimetry (ITC, S5 Fig). WT ELIC binds the agonist propylamine and the competitive antagonist acetylcholine with an effective dissociation constant (K_eff_) of 8 and 2.5 mM, respectively (Fig 3F). Whereas the affinity for the antagonist, which stabilizes the closed state of the channel, is similar in calorimetry and electrophysiology experiments [30,32], K_eff_ of the agonist is about 20-fold higher than its EC_50_ measured in two-electrode voltage clamp recordings (EC_50_ of 450 μM in the absence of Ca^2+^) [9], which suggests that the channel may not be fully activated in detergent solution. It is also noteworthy that the measured value is very close to the dissociation constant for propylamine to the resting state of 7.1 mM that was obtained from a detailed kinetic analysis of single channel recordings of ELIC [29]. To enhance binding of the agonist, we also carried out calorimetry experiments in the background of the mutant R91A located in the ligand binding site, which was previously shown to increase the potency of cysteamine, propylamine, and acetylcholine [9]. In accordance with electrophysiology, ITC experiments show that the K_eff_ values of ligands are decreased in the mutant R91A, although this effect is stronger for the agonist than the antagonist (Fig 3G). In the background of the mutant R91A, both nonactivatable mutants F116A and Y258A bind agonist and antagonist with similar K_eff_ as the single mutant R91A, thus emphasizing that the mutation has likely not affected ligand binding but instead interfered with channel activation (Fig 3H and 3I).

**Table 1:** Data collection and refinement statistics 1.

	ELIC F116A	ELIC Y258A	ELIC T28D	ELIC T28D/ligand
**Data collection**				
Space group	P2_1_	P2_1_	P2_1_	P2_1_
Cell dimensions				
*a*, *b*, *c* (Å)	105.2, 267.0, 111.0	105.3, 266.6, 110.7	105.5, 267.0, 110.2	106.8, 264.8, 108.7
*α*, *β*, *γ* (°)	90.0, 110.2, 90.0	90.0, 110.5, 90.0	90.0, 110.5, 90.0	90.0, 110.5, 90.0
Resolution (Å)	50–3.5 (3.6–3.5)	50–3.2 (3.3–3.2)	50–4.5 (4.6–4.5)	50–9.5 (9.7–9.5)
*R*_merge_ *	9.0 (77.0)	8.5 (74.1)	9.3 (189.2)	4.9 (113.7)
*I* / σ*I* *	14.2 (2.0)	15.2 (2.3)	13.1 (1.9)	14.9 (1.9)
Completeness (%) *	99.4 (99.6)	99.3 (99.2)	99.7 (99.5)	98.5 (99.8)
Redundancy *	3.4 (3.6)	3.9 (3.8)	12.0 (1.6)	6.9 (6.8)
**Refinement**				
Resolution (Å)	30–3.5	30–3.2	30–4.5	20–9.5
No. reflections	71,563	93,066	33,699	3,186
*R*_work/_ *R*_free_	21.3/25.5	22.0/24.7	23.7/27.1	27.5/28.9
No. atoms				
Protein	24,880	24,980	25,060	-
Ligand/ion	-	-	-	-
*B*-factors				
Protein	131	97.3	289	-
Ligand/ion	-	-	-	-
R.m.s. deviations				
Bond lengths (Å)	0.002	0.003	0.003	-
Bond angles (°)	0.6	0.6	0.6	-

*Values in parentheses are for highest-resolution shell.

### Mutation of a Conserved Salt Bridge

**Fig 4:**
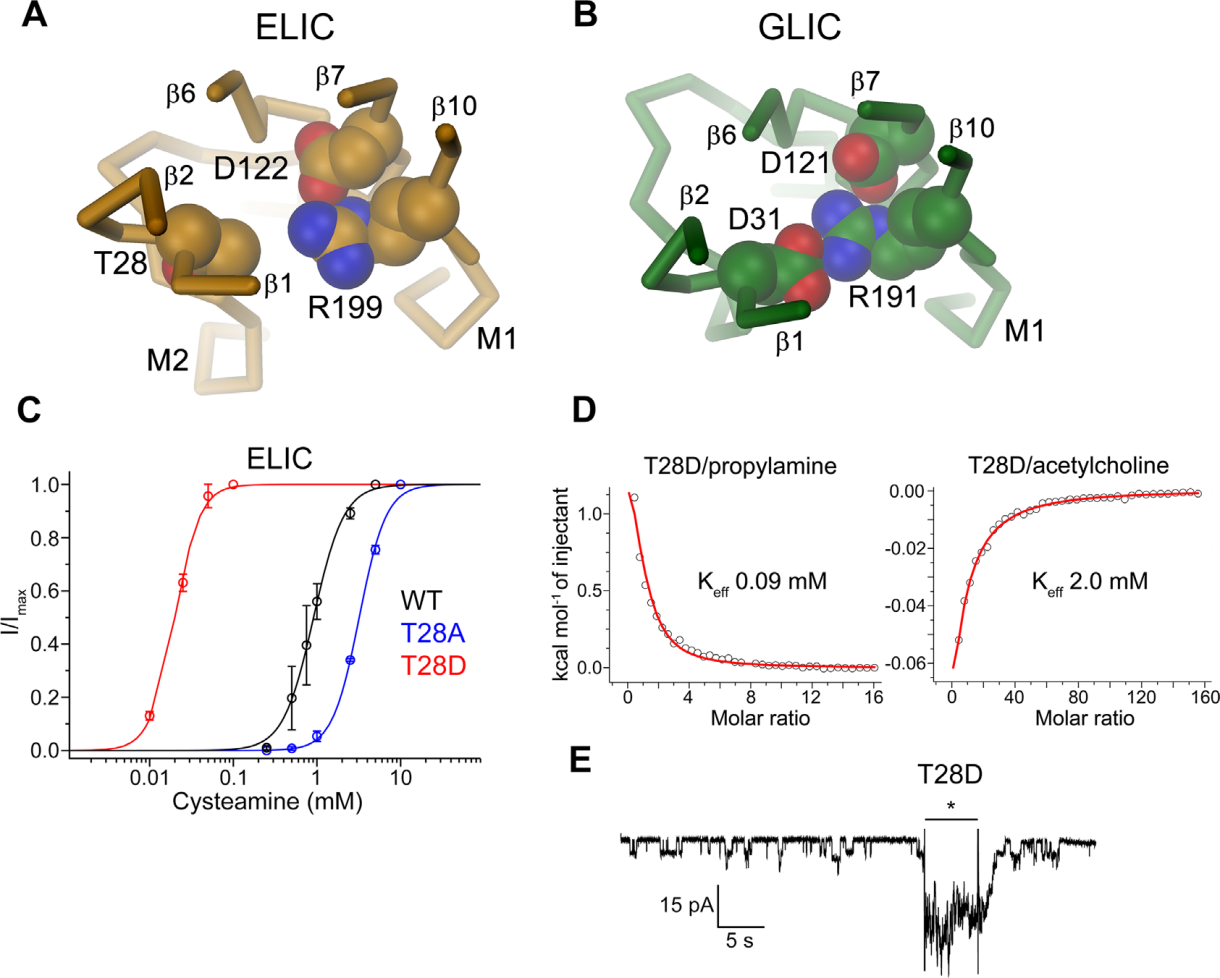
ELIC mutant with strongly increased potency for the agonist. Structure of the interaction region between the β1-β2 turn, a conserved arginine the end of β-10 and the β6-β7 loop in (A), ELIC and (B), GLIC. The view is from the extracellular side. Side-chains of selected residues forming a branched salt bridge in GLIC and their equivalent residues in ELIC are shown as CPK models. (C) Dose-response relationships of *X*. *laevis* oocytes expressing the ELIC mutants T28A and T28D measured by two-electrode voltage clamp electrophysiology. Current response of WT is shown for comparison. All currents were recorded at an outside Ca^2+^ concentration of 0.5 mM. Panels show averages of 4–6 independent measurements, solid lines show a fit to a Hill equation, errors are standard deviation (SD). (D) Agonist and antagonist binding to the ELIC mutant T28D as determined by ITC. Graphs show a fit of the integrated and corrected heat upon addition of the agonist propylamine (left) and the antagonist acetylcholine (right) to a binding isotherm (red line). (E) Patch clamp recording of the ELIC mutant T28D expressed in *X*. *laevis* oocytes. Currents were measured from excised patches in the outside-out configuration at −80 mV. Application of 20 mM cysteamine by fast solution exchange is indicated by a black bar. The recording shows considerable basal activity in the absence of the ligand. (See S1 Data for the raw data used to generate plots shown in panels C and D).

Similar to the phenylalanine in the β6-β7 loop, the mutation of an equally conserved aspartate in the same region (Asp122 in ELIC and Asp121 in GLIC) results in an apparent loss of activation in both proteins (Fig 2E and 2F). This residue is located just above the interface and forms a salt bridge with a conserved arginine (Arg199 in ELIC and Arg191 in GLIC) at the end of β-strand 10 at the boundary to the pore domain (Fig 4A and 4B and S3B Fig). The mutation of the respective arginine to alanine also causes a nonactivating phenotype in ELIC, whereas no expression of this mutant was observed in GLIC (Fig 2C–2F). In GLIC, Arg191 also interacts with Asp31 on β-strand 2, a position that, with respect to its negative charge, is conserved in many pLGIC subunits but not in ELIC where the respective residue is a threonine and the glutamate-gated chloride channel (GluCl) from *C*. *elegans* where it is a valine (S3B Fig). In GLIC, the mutant D31A is well expressed but shows no activity at pH4 (S2B Fig). In ELIC, the equivalent mutant T28A can be activated but with a 3.5-fold higher EC_50_ of agonist than WT (Fig 4C and S6A Fig). When mutating Thr28 in ELIC to aspartate, thereby introducing a negative charge that is present in many family members, the EC_50_ of channel activation shifts to a 45 times lower agonist concentration (18 μM, Fig 4C and S6B Fig). A similar increase in the affinity was observed in ITC experiments, where the agonist binds with a K_eff_ of 90 μM, a 90-fold decrease in concentration compared to WT, while the binding of the antagonist acetylcholine was unchanged (Fig 4D and S5E Fig). Patch clamp recordings already show considerable basal activity of this mutant in the absence of ligand and increased currents upon ligand application (Fig 4E), whereas no basal activity is observed in WT. The single channel conductance of the mutant is similar to WT, but the current density is lower in both two-electrode voltage clamp and patch clamp experiments (Fig 4E, S6B Fig). All experiments suggest that this mutant stabilizes a high affinity state with respect to ligand binding, and we were thus interested whether it would be sufficient to change the crystallization behavior and allow us to determine the structure of ELIC in a different conformation. While we succeeded in obtaining crystals of the T28D mutant in the same conditions, they exhibited poorer diffraction than WT and only allowed us to collect data at 4.5 Å in the absence and 9.5 Å in the presence of ligand (Table 1). The structures indicate that, despite the drastic impact on the potency of the ligand, the mutant crystallized in the familiar nonconducting conformation, which further underlines the stability of this state in a crystalline environment (S4C Fig). Collectively, our studies emphasize the importance of ionic interactions between three conserved residues located in the β1-β2 turn (GLIC Asp31, ELIC Thr28), the β6-β7 loop (GLIC Asp121, ELIC Asp122), and the pre-M1 region (GLIC Arg191, ELIC Arg199, Fig 4A and 4B) for channel activation and the stabilization of the open state.

### The Contact Region between the β1-β2 Turn and the M2-M3 Loop

**Fig 5:**
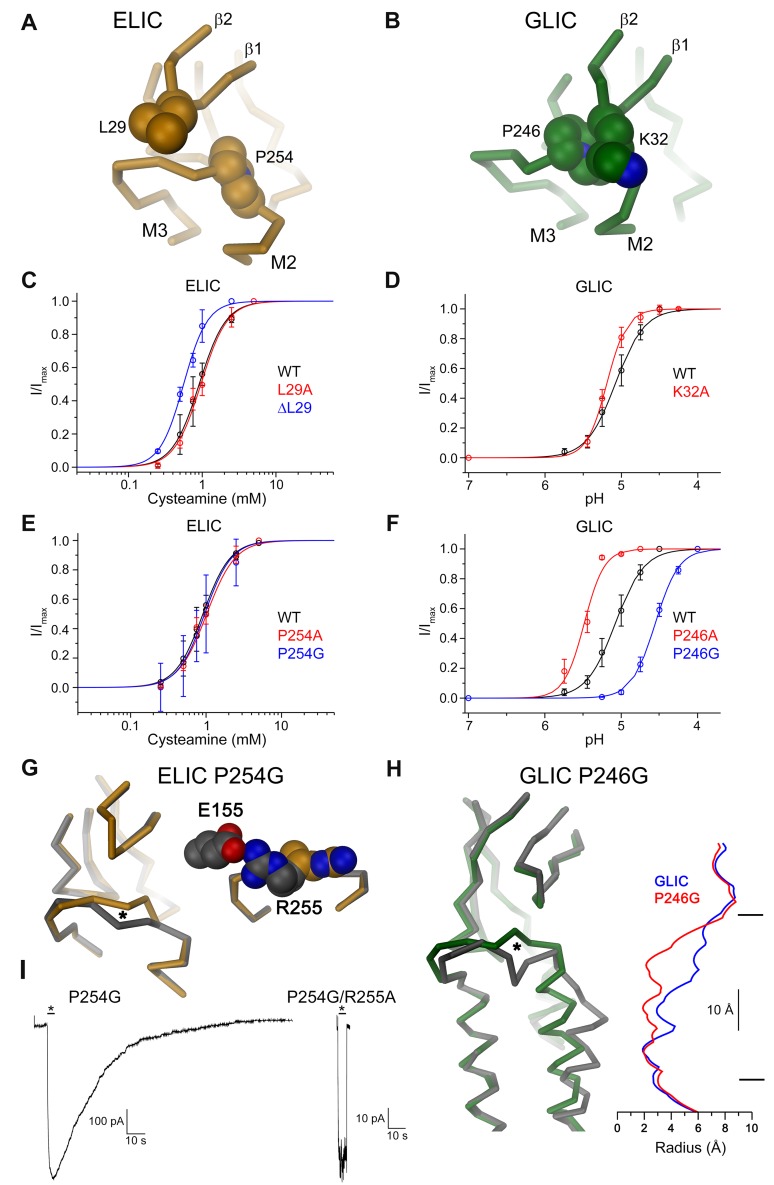
Interaction between the β1-β2 turn and the pore domain. Relationship between the residue at the tip of the β1-β2 turn and a conserved proline at the M2-M3 loop in (A), ELIC and (B), GLIC. The view is parallel to the membrane plane. Side chains of selected residues are shown as CPK models. (C–F), Dose-response relationships of *X*. *laevis* oocytes expressing mutants of either ELIC or GLIC measured by two-electrode voltage clamp electrophysiology. Current response of the respective WT is shown for comparison. For ELIC, currents were recorded at an outside Ca^2+^ concentration of 0.5 mM. Panels show averages of 3–5 independent measurements, solid lines are a fit to a Hill equation, errors are SD. Mutants of the β1-β2 turn: (C), ELIC mutant L29A and the channel carrying a deletion of Leu29 (ΔL29). (D), GLIC mutant K32A. Mutants of the M2-M3 loop: (E), ELIC mutants P254A and P254G. (F), GLIC mutants P246A and P246G. For ELIC P254G, ligand was not removed following application due to the very slow deactivation of the channels. (G) Refined structure of the ELIC mutant P254G. Left, Cα-trace of part of a single subunit of P254G (grey) showing the domain interface superimposed on ELIC WT (orange). An asterisk marks the site of mutation. Right, structure of the M2-M3 loop of the mutant P254G and of WT with side chains of R255A and E155 from the adjacent subunit shown as CPK model. (H), Structure of the GLIC mutant P246G. Left, Cα-trace of part of a single subunit of P246G (grey) showing the domain interface and part of the pore region superimposed on GLIC WT (green). An asterisk marks the site of mutation. Right, Pore radius of GLIC (blue) and P246G (red) along the channel axis. The membrane boundary is indicated. (I) Macroscopic currents recorded from representative patches of the ELIC mutant P254G (left) and the double mutant P254G/R255A (right) upon fast application and washout of agonist. Currents were recorded from membrane patches of the respective ELIC mutants expressed in HEK293 cells in response to application of 25 mM propylamine. The difference in the deactivation rate in both mutants is apparent. (See S1 Data for the raw data used to generate plots shown in panels C-F).

At the tip of the GLIC β1-β2 turn, immediately adjacent to Asp31, Lys32 interacts with a strictly conserved proline in the M2-M3 loop (Pro254 in ELIC and Pro246 in GLIC, Fig 5A and 5B). This interaction is also observed in the presumably open structures of GluCl, the GlyR, and the structures of a homopentameric GABA_A_ receptor. Conversely, this interaction is not formed in the structures of ELIC, the ligand-free GluCl, the antagonist-bound GlyR and the structure of the 5-HT_3_ receptor, where the contact is broken (S3C Fig). We thus suspected that this interaction might play an important role for the relay of conformational changes from the extracellular domain to the pore [14,28]. In all pLGICs of known structure, the interaction between the tip of the β1-β2 turn and the pore domain is mediated by the protein backbone, whereas the side chain of the respective residue points towards the channel lumen (Fig 5A and 5B and S3C Fig). In contrast to other residues in the interaction interface, this position is not conserved and contains a lysine in GLIC and the 5-HT_3_ receptor and a hydrophobic amino acid in ELIC, GluCl and the GABA_A_ receptor. When expressed in *X*. *laevis* oocytes, the respective alanine mutants in ELIC and GLIC can still be activated with similar EC_50_ values as the respective WT (Fig 5C and 5D, S6C and S6D Fig), although with a lower maximal current response (S2, S6C and S6D Figs). Remarkably, even a deletion mutant of L29 in ELIC showed a comparable activation pattern (Fig 5C and S6E Fig). Mutations of the conserved proline in the M2-M3 loop to alanine (P246A in GLIC and P254A in ELIC) resulted in channels that, apart from a small shift in the EC_50_ in the case of GLIC, show robust activation with similar properties as WT (Fig 5E and 5F, S6F and S6G Fig). In line with the comparably small effect in functional experiments, the crystal structure of the GLIC mutant P246A is virtually identical to WT (S7A and S7B Fig, Table 2). To avoid residual side chain interactions with the ligand-binding domain that may still be present after replacing the proline by alanine, we next investigated the mutation of the respective proline residue in both channels to glycine. In ELIC, the mutation P254G has a small but significant effect on the structure, which overall shows the frequently observed nonconducting conformation. The reorganization of the well-structured M2-M3 loop indicates a change in the conformational properties of this region (Fig 5G, S4D Fig, Table 2). The equivalent mutation P246G in GLIC results in large rearrangements when compared to WT. The structure determined at 3.2 Å shows a molecule with small differences throughout most of the protein except for the M2-M3 loop and the pore-lining helix M2, which both have undergone major conformational changes (Fig 5H, S7C Fig, Table 2). The introduced conformational freedom upon replacement of the restrained amino acid proline with the flexible glycine has resulted in the rearrangement of the M2-M3 loop and the unfolding of the C-terminal part of M2. The remainder of the helix has collapsed towards the pore axis as to maximize the hydrophobic interactions of residues at the extracellular part leading to a structure, which, most probably, prevents the permeation of ions (Fig 5H, S7D and S7E Fig). In contrast to the large conformational change in the extracellular part of the helix, its conformation at the intracellular half remained unchanged. Remarkably, this pore conformation is very similar to structures of GLIC obtained from cysteine crosslinking of residues at the domain interface, which were previously assigned to a locally closed conformation of the ion conduction path [33], the structures of two nonactivating mutants in the M2-M3 loop [34] and a recent structure of GLIC at neutral pH [20]. In this locally closed conformation, an interaction of a histidine residue in the pore-forming helix M2 with the backbone of the neighboring helix M3 established in the low pH crystal structure of GLIC is broken (S7F Fig). It is noteworthy that the histidine is in a position that in other family members of known structure is predominantly hydrophobic, and that it was previously proposed to be involved in the pH-dependent activation of GLIC [35,36].

**Table 2:** Data collection and refinement statistics 2.

	ELIC P254G	GLIC P246A	GLIC P246G
**Data collection**			
Space group	P2_1_	C2	C2
Cell dimensions			
*a*, *b*, *c* (Å)	105.0, 266.6, 110.7	177.7, 133.9, 159.8	180.4, 134.2, 160.8
*α*, *β*, *γ* (°)	90.0, 109.3, 90.0	90.0, 101.1, 90.0	90.0, 102.0, 90.0
Resolution (Å)	50–3.3 (3.4–3.3)	50–3.3 (3.4–3.3)	50–3.2 (3.3–3.2)
*R*_merge_ *	11.5 (90.1)	10.1 (77.3)	12.2 (95.6)
*I* / σ*I* *	12.2 (2.5)	13.1 (2.4)	9.3 (1.8)
Completeness (%) *	99.8 (99.8)	99.6 (99.6)	98.3 (82.8)
Redundancy *	5.4 (5.4)	5.3 (5.4)	4.4 (4.3)
**Refinement**			
Resolution (Å)	30–3.3	30–3.3	30–3.2
No. reflections	85,767	55,060	60,831
*R*_work/_ *R*_free_	22.1/25.2	24.6/28.0	24.7/25.8
No. atoms			
Protein	25,020	12,595	12,590
Ligand/ion	-	-	-
*B*-factors			
Protein	109	97	101
Ligand/ion	-	-	-
R.m.s. deviations			
Bond lengths (Å)	0.003	0.003	0.004
Bond angles (°)	0.64	0.65	0.78

*Values in parentheses are for highest-resolution shell.

Despite the strong impact of the mutation on the structure, both proteins can still be activated (Fig 5E and 5F, S6G–S6J Fig). When studied by two-electrode voltage clamp electrophysiology, the GLIC mutant P246G shows dose-dependent channel activation with an EC_50_ that is shifted by 0.5 pH units towards higher proton concentrations (Fig 5F and S6H Fig). In ELIC, the mutant P254G shows agonist-induced currents with a similar EC_50_ as WT but with a slower activation and an unusually slow deactivation of the channel upon washout of the ligand (Fig 5E, S6I Fig). This behavior can be observed in two-electrode voltage clamp recordings, where the activity of the protein after a change to ligand-free solution decays slowly (S6J Fig), and it becomes even more pronounced in macroscopic recordings of excised outside-out patches (Fig 5I, S8A–S8C Fig). The cause for this unusual deactivation phenotype was revealed in the structure of the P254G mutant of ELIC. In this structure, electron density between Arg255 on the M2-M3 loop and Glu155 located in the β8-β9 loop of the extracellular domain of an adjacent subunit indicates the formation of a strong ionic interaction that is absent in WT and that may stabilize the open conformation of the pore (Fig 5G, S4D Fig). In the background of the mutation R255A, the kinetics of channel deactivation of the P254G mutant becomes similar to WT, thus confirming the role of the interaction for the unusual functional behavior (Fig 5I and S8D Fig). The single mutation of R255A behaves similar to WT but shows faster activation and deactivation kinetics and an increased rate of desensitization (S8E–S8G Fig). Thus, to our surprise, the mutation of a conserved proline in the M2-M3 loop to glycine still promotes activation in both channels, ELIC and GLIC. These results are in contrast to the much more drastic effects that are observed in mutants of other residues at the domain interface, including several positions in the same region, where even the truncation of the side chain to alanine has apparently prevented channel activation.

## Discussion

We have used a mutagenesis approach to investigate the role of the domain interface of two prokaryotic pLGICs for the transduction of conformational changes from the extracellular to the pore domain. Our study has revealed a pattern of corresponding residues in ELIC and GLIC that, if mutated to alanine, had a similar effect on either channel. Remarkably, whereas mutations in several positions have apparently prevented activation in both proteins, none of the investigated alanine mutations showed detectable basal activity in the absence of agonists. Our results suggest that the respective side chain truncations may have either stabilized a closed conformation of the channel, where the energy of ligand binding is no longer sufficient for activation, or alternatively, that they have interfered with the coupling of both domains and that the pore region that is uncoupled from the extracellular domain resides in a stable nonconducting conformation. In all cases, the mutations likely did not interfere with ligand binding but instead impeded gating, as suggested by calorimetry experiments of two nonactivating mutants of ELIC, which showed WT-like binding properties of agonists and antagonists (Fig 3H and 3I). In case of WT, the observed agonist binding affinity is lower than expected from the EC_50_ value measured by electrophysiology [9] and instead matches the binding affinity to the resting state obtained from single channel analysis [29] (Fig 3F). It thus appears that in detergent solution, ELIC resides in a single conformation that, with respect to ligand binding, resembles a resting state. Assuming that the conformation that is probed by calorimetry is also observed in the crystal, since in both cases the protein is solubilized in the same detergent, it is unlikely that the structure of ELIC represents a desensitized state with high affinity for the ligand. The conformational rigidity of solubilized ELIC is in accordance with the fact that all currently available structures show the same nonconducting conformation of the protein, irrespectively of whether agonist is bound or mutations were introduced that have stabilized the open state as it is the case for the mutations T28D or P254G. This behavior is in line with previous observations for ELIC [9,37]. Additionally, a reduced conformational freedom in detergent solution was observed in an electron paramagnetic resonance (EPR) spectroscopy study of GLIC [38], which suggests that both proteins may require a lipid environment for full activation. Whereas the predominantly local effects of most mutations, which modulate the open to closed equilibrium of the channel, resembles the behavior of eukaryotic receptors [39], the mutation of a conserved proline (Pro254) to glycine in ELIC resulted in the formation of novel interactions between residues apart from the site of mutation that were not present in WT, causing an unusual functional phenotype that would have been difficult to explain in the absence of structure (Fig 5G).

Our studies show that mutations with a nonactivating phenotype predominantly cluster in two regions of the protein, the β6-β7 loop of the extracellular domain and the M2-M3 loop of the pore. The results are in accordance with a previous investigation of two mutations in the M2-M3 loop of GLIC [34], and they overall mirror the functional behavior of eukaryotic pLGICs [40–43]. Differences between pro- and eukaryotic channels may originate from an altered energetic relationship between distinct states, and effects may generally be less pronounced if mutations only concern one or two subunits of a heteropentameric receptor. An early study has identified a mutation in the M2-M3 loop of the homopentameric α7 nAChR, which prevents activation but not ligand binding [44]. A similar phenotype was found for a mutation of the equivalent residue of the GlyR causing Startle disease, as well as by an additional mutation located two residues upstream [45]. Based on these observations, an involvement of the M2-M3 loop in gating has been proposed [44–46]. Similarly, our study has shown that mutations of the corresponding positions in the two prokaryotic channels (T248A, Y250A in GLIC and L256A, Y258A in ELIC) have interfered with activation but not ligand binding, as suggested by the calorimetry experiments of the ELIC mutant Y258A. The same region was also investigated in the hetero-pentameric muscle nAChR. Based on the effect of mutations on the equilibrium and kinetics of the open to closed transition, a prominent role of the M2-M3 loop of the α-subunits on the activation of the channel was postulated, but in this case the positions with the strongest phenotype differ from the residues identified in this study [39,47,48]. Whereas the kinetics of ELIC and GLIC is comparably slow [9,29,49], we found that the point mutation R255A in the M2-M3 loop of ELIC not only accelerated activation and deactivation but also increased the rate of desensitization (S8E–S8G Fig). In that respect, it is interesting to note that alanine is found in the same position of the fast desensitizing α7 nAChR and that a mutation of the corresponding residue of the GlyR causes Startle disease [50]. Since in a different study the domain interface was shown to influence the desensitization rate of homomeric pLGICs [51], it appears that the same region determines activation and desensitization of the channels.

Like the M2-M3 loop, the β6-β7 loop of eukaryotic pLGICs has also been proposed to play a critical role in channel activation. Mutations in equivalent positions that interfered with activation in both prokaryotic channels decreased the agonist response of the α1 GlyR and abolished the potentiation of currents by general anesthetics [52]. In a different study, the activation in chimeras of the α7 nAChR and the GlyR was enhanced by point mutations of residues of the β6-β7 loop that are in equivalent positions as nonactivating mutants in ELIC and GLIC [53]. A strong effect of mutations on the gating equilibrium constant was also found in the α-subunit of the nAChR [39,43,47], and strong energetic coupling of two conserved phenylalanines of the β6-β7 loop to the M2-M3 loop was proposed based on mutant cycle analysis [54].

**Fig 6:**
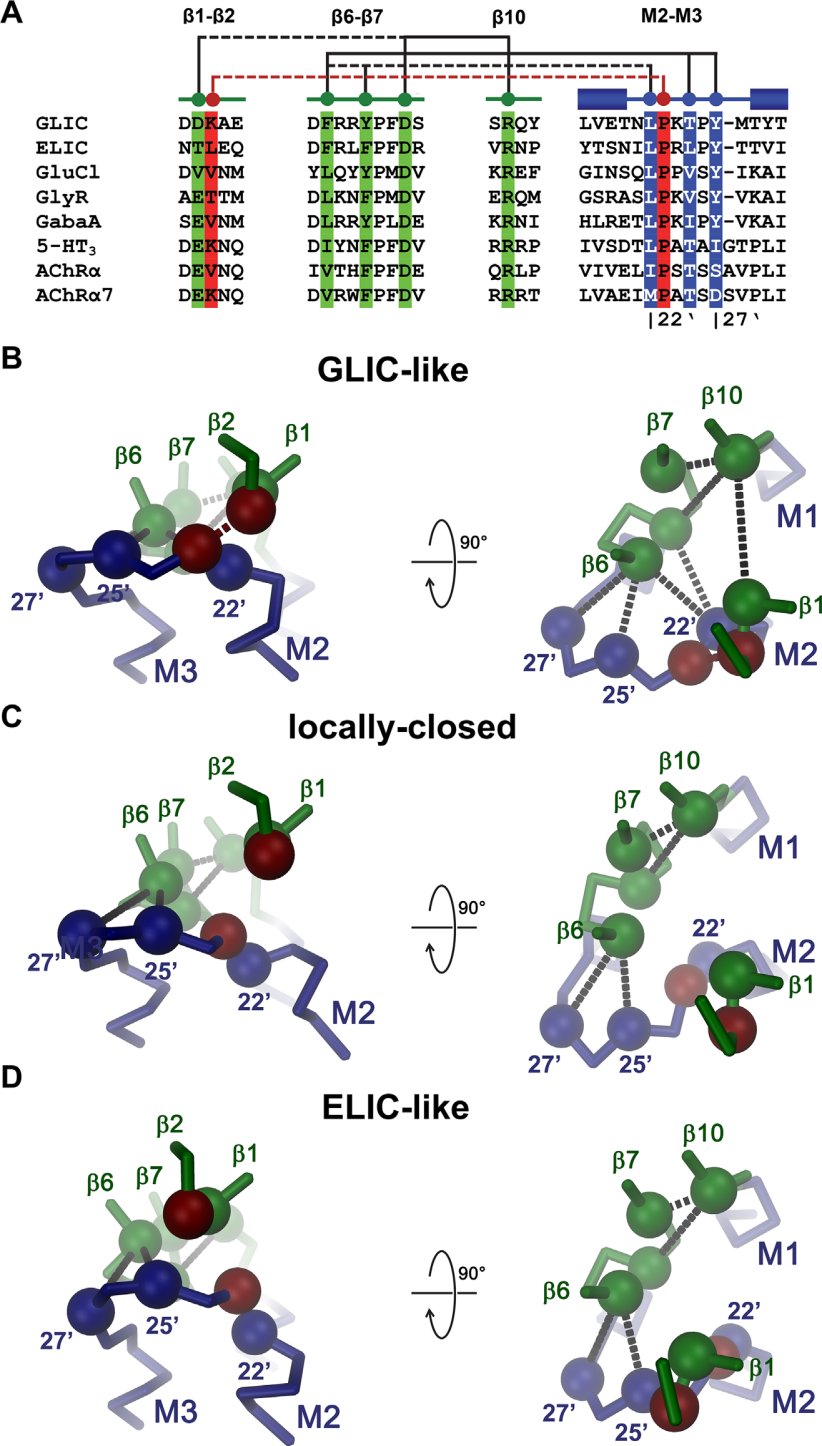
Role of the domain interface in channel activation. Interactions between residues at the domain interface in different channel conformations. (A) Sequence alignment of regions of the domain interface. Selected residues and their interactions are highlighted and schematically depicted above. Solid lines indicate interactions that are present in all conformations and dashed line interactions that are only formed in the presumably open GLIC-like conformation. Numbering below the sequences indicates the position of residues in the pore. Sequences are GLIC (UniProt: Q7NDN8), ELIC (GB: WP_013319743), GluCl (GB: AAA50785.1), GlyR (GB: NP_571477.1), Gaba_A_ (GB: M82919), 5-HT3 (GB: NM_001099644.1), AChRα (UniProt: P04756), AChRα7 (UniProt: P49582). Cα-trace of the interface of a single subunit of (B) GLIC in a presumably open conformation (GLIC-like), (C) GLIC at pH 7.0 (locally-closed), (D) ELIC (ELIC-like). The view is from within the membrane (left) and from the extracellular side (right). The pore domain is colored in blue and the extracellular domain in green. The positions of selected residues whose mutation exerts a strong effect on activation in ELIC and GLIC are shown as spheres. The residue at the tip of the β1-β2 turn and a conserved proline in the M2-M3 loop are colored in red. Selected interactions (as in A) are indicated by dashed lines.

In ELIC and GLIC, a nonactivating phenotype was found for mutations of a conserved aspartate of the β6-β7 loop (Asp122 in ELIC and Asp121 in GLIC) that interacts with an equally conserved arginine in the pre-M1 region (Arg199 in ELIC and Arg191 in GLIC). Mutations of the corresponding aspartate also interfered with activation in the nAChR [43,55], the 5-HT_3_ receptor [56] or the GlyR [57]. In GLIC, the pre-M1 arginine bridges the β6-β7 loop with the β1-β2 turn by interaction with a negatively-charged residue (Asp31) that is found in most pLGIC subunits (Fig 6A, S3B Fig). The mutation of this residue to alanine prevents activation of GLIC, whereas the mutation of a threonine residing at the equivalent position in ELIC (T28A) appears to remain functional, although with decreased potency of the agonist (Fig 4C, S2 Fig). The role of this interaction in channel activation and the destabilization of the resting state is underlined by a mutation of the respective threonine to aspartate in ELIC, which strongly increases the potency for the agonist and where the channel shows increased basal activity, which is not observed in WT (Fig 4). This basal activity demonstrates that ELIC can, in principle, also open in the absence of agonist and thus underlines the validity of the MWC model also for this channel. Equivalent ionic interactions have previously been investigated in the nAChR and other pLGICs [54–56,58,59]. In one study, they were postulated to be part of a molecular pathway that plays an important role in the relay of signals from the extracellular domain to the pore region, thereby connecting ligand binding to gating [54,59]. Since similar but smaller effects were found in a different study, the central importance of this ionic interaction for channel activation was questioned [58], and it was instead proposed that the total charge of the interface rather than specific pairwise interactions may govern channel activation in the nAChR [55]. Contrary to our previous expectation [14,28], conformational changes appear not to be predominantly transduced via an interaction of the tip of the β1-β2 turn to a conserved proline in the M2-M3 loop of the pore domain (Pro254 in ELIC and Pro246 in GLIC), as mutations of this residue to alanine and glycine still permit channel activation. This is remarkable since the equivalent proline was proposed to play a prominent role in the early events of gating in the nAChR [39,48], and since in several structures of different pLGICs assigned to potentially open conformations, this interaction is present, whereas it is broken in presumably nonconducting conformations (S3C Fig). A coupling of the β1-β2 turn to a proline of the M2-M3 linker in the nAChR was previously proposed based on a model of the receptor from electron microscopy data at 4 Å [10,54,59]. However, due to a mismatch in the structural interpretation of this region, this position does not coincide with the residue investigated in this study.

The structures of different pLGICs at high resolution [12–21] provide a framework for the comprehension of the results of this study. With respect to the transmembrane domain, most known structures cluster around three distinct conformations: A presumably conducting state of the pore, which has initially been observed for a low pH crystal form of GLIC [14,15], is shared by GluCl and the GlyR in complex with their agonists and the allosteric modulator ivermectin [12,16], as well as the GABA_A_ receptor [17] (S9 Fig). It is still debated whether these structures correspond to conducting, partially conducting, or even desensitized states [22]. A structure of the GlyR in complex with glycine has a larger pore diameter at the intracellular part of the transmembrane domain but shares a very similar pattern of interactions at the domain interface [16]. Conformations resembling the nonconducting state of ELIC were later observed for GluCl crystallized in the absence of ortho- and allosteric agonists, a conformation of the ligand-free protein in complex with a bound lipid [19], and for the GlyR bound to its competitive antagonist strychnine [12,21] (S9 Fig). This is remarkable in light of the controversy concerning the relationship of the ELIC structure to a resting conformation of the receptor [22,37,38,60]. The structure of the 5-HT_3_ receptor is in between the two previously described states but closer to the GLIC-like conformation [18] (S9 Fig). Another distinct nonconducting conformation of the pore region was observed in a high pH crystal form of GLIC [20] and in several mutants of the same channel [33,34], including the structure of the mutant P246G determined in this study (S9 Fig). This third conformation has thus far only been observed in GLIC, a member of the family that is activated by protons, and it remains to be shown whether a similar conformation of the pore region can also be adopted by other family members. The fact that side chains that are truncated in nonactivating mutants point into a common core suggests that the mutation may have disrupted a critical interaction (Fig 2A and 2B and S3A Fig). These interactions appear to be most extended in the presumably open GLIC-like conformations (Fig 2A and Fig 6). In these cases, the interface between the extracellular domain and the pore region is tightly packed, residues from the M2-M3 loop are in close contact with residues of the β6-β7 loop, and in several structures a negatively charged residue in the β1-β2 turn interacts with a conserved arginine in the pre-M1 region and an equally conserved proline in the M2-M3 loop (Fig 6A and 6B, S3B and S3C Fig). This network is partially disrupted in the two nonconducting conformations, which might explain why several mutations in the interaction interface stabilize a closed state of the channel. In the locally-closed high pH structure of GLIC, the distance between the β1-β2 turn and the arginine in the pre-M1 region has increased and, due to a change of the conformation of the M2-M3 loop leading to a collapse of the pore-forming helix M2, the contact to the proline in the respective region is broken (Fig 6C, S3C Fig). This conformational change also causes an interruption of interactions between residues in the N-terminal part of the M2-M3 loop with the β6-β7 loop, whereas the interactions of the residues in the region preceding M3 appear less affected (Fig 6C). In the ELIC-like conformations, a similar pattern of interactions is observed, but in this case, the disruption of interdomain contacts is due to a concerted move of helices M2 and M3 while preserving the conformation of the M2-M3 loop (Fig 6D). The accompanying rearrangement of the β1-β2 turn of the extracellular domain weakens its interaction with the pre-M1 region and disrupts the contact to the proline in the M2-M3 loop. In both nonconducting conformations, the interactions with the C-terminal part of the M2-M3 loop with the β6-β7 linker remain intact, and the observed nonactivating phenotype of respective mutations (F116A and Y258A in ELIC and F115A and Y250A in GLIC) could thus originate from an interruption of the coupling between the extracellular domain and the pore region.

Despite the plethora of structural information, definitive assignments of observed conformations to functional states of the receptors and the resulting activation mechanisms are still controversial [22]. It is thus interesting to observe that, regardless of the difference of agonists, highly conserved and closely interacting residues at the domain interface of ELIC and GLIC exert similar effects on activation in both prokaryotic ion channels. Critical interactions involve residues of the M2-M3 loop, the pre-M1 region and the β1-β2 turn that all contact the β6-β7 loop, whereas a direct interaction between the β1-β2 turn and the M2-M3 loop appears expendable. As in eukaryotic receptors, mutations at the interface predominantly affect the close to open equilibrium of the pore. Our results thus suggest that there is a common pathway for signal transduction in both proteins that, regardless of differences in the detailed energetic relationships between pro- and eukaryotic receptors, appears to be conserved within the entire family.

## Materials and Methods

### Expression Constructs

All expression constructs were cloned into vectors that were modified to be compatible with FX cloning [61]. For expression in *X*. *laevis* oocytes, WT and mutant open reading frames of ELIC and GLIC preceded by the signal sequence of the chicken α7 nAChR were cloned into a modified pTLN vector [62]. For surface expression analysis, the constructs contained an additional hemagglutinin-tag (HA-tag) attached to the N-terminus of the respective protein. For expression in human embryonic kidney 293 (HEK293) cells, the respective genes preceded by the signal sequence of the chicken α7 nAChR were cloned into a modified pcDNA3.1 vector (Invitrogen). For expression and purification in *E*. *coli*, the respective genes were cloned into a modified pET26b vector (Novagen) as constructs of the respective channels preceded by a fusion protein consisting of a pelB signal sequence, a His_10_-tag, maltose-binding protein and a human rhinovirus (HRV) 3C protease cleavage site.

### Two-Electrode Voltage Clamp Recording and Surface Expression Analysis in *Xenopus* Oocytes

*X*. *laevis* oocytes were obtained either from Ecocyte or from an in-house facility. Animal procedures and preparation of oocytes followed standard procedures and were in accordance with the Swiss Cantonal and Federal legislation relating to animal experimentation. Plasmid DNA containing the genes coding for the respective constructs for expression in *X*. *laevis* oocytes were linearized by MluI, and capped mRNA was transcribed with the mMessage mMachine kit (Ambion) and purified with the RNeasy kit (Qiagen). 10–200 ng of mRNA was injected into defolliculated *X*. *laevis* oocytes, which were subsequently incubated in Barth’s solution (88 mM NaCl, 1 mM KCl, 1 mM CaCl_2_, 0.33 mM Ca(NO_3_)_2_, 0.82 mM MgSO_4_, 10 mM Na-Hepes (pH 7.4) and 50 μg / ml Gentamycin) and stored at 16°C. One to three days after injection, two-electrode voltage clamp measurements were performed at 20°C (OC-725B, Warner Instrument Corp.). For ELIC, maximal currents were recorded in a bath solution containing 10 mM Hepes (pH 7), 130 mM NaCl, 2 mM KCl, 0.5 mM CaCl_2_ and either 5 mM or 25 mM Cysteamine. For GLIC, the maximal currents were recorded in a bath solution containing 10 mM Citrate (pH 4), 130 mM NaCl, 2 mM KCl, 1.8 mM CaCl_2_ and 1 mM MgCl_2_. Dose-response experiments were carried out at agonist concentrations indicated in the respective figures. Voltage was clamped at −40 mV, and data was filtered at 20 Hz unless stated otherwise. Surface expression in constructs containing an HA-tag was assayed after electrophysiological characterization as described [63]. For that purpose, the oocytes were placed in a 96-well plate (TPP) and incubated in ND96 solution (93.5 mM NaCl, 2 mM KCl, 1.8 mM CaCl_2_, 2 mM MgCl_2_ and 10 mM Hepes, pH 7.4) containing 1% BSA for at least 30 min. All steps were carried out at 4°C with the same buffer unless mentioned otherwise. Oocytes were subsequently transferred into buffer containing 1 μg/ml rat monoclonal anti-HA antibody (3F10, Roche) for 1 h, washed 3 times and incubated with buffer containing 0.16 μg/ml horseradish peroxidase (HRP) coupled to a secondary antibody (HRP-conjugated goat anti-rat F(Ab)2 fragments, Jackson) for 30–60 min. The oocytes were washed 5 times with ND96 solution and subsequently transferred to a white 96-well plate (flat bottom, Nunclon Delta Surface). The solution was aspirated, 30 μl of Super Signal ELISA femto solutions 1 and 2 (Pierce) was added, and luminescence was quantitated with a Tecan infinite M1000 plate reader.

### Patch Clamp Recording in *X*. oocytes

*X*. *laevis* oocytes were transferred to a hyperosmotic solution to manually remove the vitelline layer. Excised membrane patches were subsequently recorded in the outside-out configuration 3–5 d after injection of mRNA with an Axopatch 200B amplifier (Axon Instruments) at 20°C at −80 mV. Data was sampled at 20 kHz and filtered at 2 kHz and analyzed using Clampfit (Axon Instruments, Inc.). Bath solutions contained 10 mM HEPES, pH 7.0, 150 mM NaCl, 0.2 mM CaCl_2_ and indicated concentrations of ligands. Electrodes had a resistance of 3–5 MΩ. Pipette solutions contained 150 mM NaCl, 10 mM EGTA, 5 mM MgCl_2_ and 10 mM HEPES, pH 7.0. Bath electrodes were placed in 1 M KCl solution connected to the bath solution by Agar bridges. Freshly prepared agonist solutions were applied to the patch using a stepper motor (SF77B Perfusion fast step, Warner).

### Patch Clamp Recording in HEK293 Cells

HEK293 cells (American Type Culture Collection-CRL-1573;LGC Promochem) were maintained at 37°C in a 95% air/5% CO_2_ incubator in DMEM supplemented with 0.11 g/l sodium pyruvate, 10% (v/v) heat-inactivated fetal bovine serum, 100 U/ml penicillin G, 100 μg/ml streptomycin sulfate, and 2 mM L-glutamine (Invitrogen). Cells (passaged every 2 d, up to 30 times) were plated and transfected by calcium phosphate-DNA coprecipitation [64], with a total amount of DNA of 3 μg/dish (82% ELIC and 18% eGFP DNA, both subcloned in pcDNA3.1). Cells were bathed in an extracellular solution containing 150 mM KCl, 0.2 mM CaCl_2_ and 10 mM HEPES, pH 7.4. Patch pipettes were pulled from thick-walled borosilicate glass (GC150F; Harvard Apparatus) and fire polished to a resistance of 8–12 MΩ. Intracellular solution contained 150 mM KCl, 0.5 mM CaCl_2_, 5 mM EGTA and 10 mM HEPES, pH 7.4. Agonist-evoked currents were recorded at 20°C with an Axopatch 200B amplifier (Molecular Devices) from outside-out patches at −50 mV. No correction for junction potential was applied (calculated value 0.2 mV). Data was sampled at 10 kHz and filtered at 1 kHz and analyzed using Clampfit (Axon Instruments, Inc.). All concentration jumps were performed using a piezo stepper (Siskiyou) with an application tool made from theta tube glass (Hilgenberg; final tip diameter, 150 μm). Agonist solutions were freshly prepared before measurements.

### Protein Expression and Purification

ELIC WT and point mutants were expressed and purified as described [9,13]. BL21-DE3 cells transformed with a pET26b vector carrying the respective expression constructs of ELIC were grown in M9 minimal medium containing 50 mg/l kanamycin at 37°C to an OD_600_ of 1.0 and subsequently cooled to 20°C. Expression was induced by addition of 0.2 mM IPTG and carried out overnight. BL21-DE3 cells transformed with a pET26b vector carrying the respective expression constructs of GLIC were grown at 37°C in TB medium containing 50 mg/l kanamycin to an OD_600_ of 1.6–1.8. Expression was induced by addition of 0.2 mM IPTG overnight at 20°C. All following steps were performed at 4°C. ELIC was extracted from isolated membranes in a buffer containing 1% n-Undecyl-β-D-Maltoside (UDM, Anatrace, Inc.) and further purified in buffers containing 0.145% UDM. GLIC was extracted from isolated membranes in a buffer containing 1% n-Dodecyl-β-D-Maltoside (DDM, Anatrace, Inc.) and further purified in buffers containing 0.044% DDM. Both proteins were purified by Ni-NTA chromatography (Qiagen) and digested with HRV 3C protease to cleave the His_10_-MBP fusion tag. His_10_-MBP and 3C protease were subsequently removed from solution by binding to Ni-NTA resin and the flow-through was concentrated and subjected to gel-filtration on a Superdex 200 column (GE Healthcare). The protein peak corresponding to the ELIC pentamer was pooled, concentrated to 10 mg/ml and used for crystallization and ITC. The protein peak corresponding to the GLIC pentamer was pooled and concentrated to 10 mg/ml, and used for crystallization.

### Crystallization and Structure Determination

Both proteins were crystallized in sitting drops at 4°C as described [9,13]. ELIC containing additional 0.5 mg/ml *E*. *coli* polar lipids (Avanti Polar Lipids, Inc.) was mixed in a 1:1 ratio with reservoir solution composed of 200 mM (NH_4_)_2_SO_4,_ 50 mM ADA, pH 6.5 and 10%–13% (w/v) PEG4000. GLIC containing additional 0.5 mg/ml *E*. *coli* polar lipids was in mixed in a 1:1 ratio with reservoir solution composed of 225 mM (NH_4_)_2_SO_4_, 50 mM sodium acetate, pH 4.0 and 9%–12% (w/v) PEG 4000. The crystals were cryoprotected by transfer into solutions containing additional 30% ethylene glycol. All data sets were collected on frozen crystals on the X06SA beamline at the Swiss Light Source (SLS) of the Paul Scherrer Institute (PSI) on a PILATUS detector (Dectris). The data were indexed, integrated, and scaled with XDS [65] and further processed with CCP4 programs [66] (Tables 1 and 2). The structure of mutants was determined by molecular replacement in PHASER [67] using either the ELIC pentamer in a P4_3_ crystal form (2YN6) or the GLIC pentamer (3EHZ) as search model. The models were rebuilt in Coot [68] and refined maintaining strong NCS restraints in PHENIX [69]. R and R_free_ were monitored throughout. R_free_ was calculated by selecting 5% of the reflection data in thin slices that were selected for the initial datasets of ELIC and GLIC and that were omitted in refinement. For low resolution data of the ELIC mutant T28D, refinement was restricted to rigid body refinement followed by few cycles of restrained positional and group b-factor refinement. The pore radii were calculated with HOLE [70].

### ITC

Binding of the agonist propylamine and the antagonist acetylcholine to ELIC was measured by ITC with a MicroCal ITC200 system (GE Healthcare). The syringe was loaded with agonist solution containing between 30–37 mM propylamine or acetylcholine dissolved in measurement buffer (25 mM Hepes, pH 7.0, 150 mM NaCl and 0.9 mM UDM). The sample cell was loaded with 300 μl of purified ELIC in measurement buffer at a concentration between 80–110 μM. Agonist was applied by sequential injections of 2 μl aliquots followed by a 180 s equilibration period after each injection. The data was recorded at 4°C. For analysis, the heat released by each injection was integrated, and the background was subtracted with NITPIC [71]. The background-corrected data was analyzed by a fit to a single-site binding isotherm with the Origin ITC analysis package. ITC experiments were performed at least twice for each protein, with similar results.

## Supporting Information

S1 Data Numerical data used in preparation of Figs 2C, 2D, 3F, 3G, 3H, 3I, 4C, 4D, 5C, 5D, 5E, 5F, S2A, S2B, S6B, S6E, S6H, S6J, S8G and S9D. (XLSX)

S1 Fig View of the domain interface. Stereo view of the domain interface of ELIC (A) and GLIC (B). The view is from within the pore parallel to the membrane plane. The respective proteins are displayed as Cα-trace, residues mutated in this study as sticks. Carbon atoms of different regions are shown in unique colors as indicated on the bottom. Selected secondary structure elements are labeled; apostrophes refer to adjacent subunit.
(TIF)

S2 Fig Surface expression and agonist response of alanine mutants. Surface expression levels of alanine mutants of ELIC (A) and GLIC (B) determined by ELISA. Values are averages of 4–6 experiments. Background from empty oocytes was subtracted and signal was normalized to WT. Errors are SEM. The protein sequence is indicated above each chart with nonactivating mutations colored in red. The residue number of selected residues is indicated. Current response at high agonist concentration (ELIC: 25 mM cysteamine, GLIC: pH 4) of representative oocytes expressing the indicated mutants are shown above the respective chart for ELIC and below for GLIC. Currents were either recorded at −40 mV or scaled to the expected value at −40 mV assuming a linear macroscopic conductance. (See S1 Data for the raw data used to generate plots shown in panels A and B).
(TIF)

S3 Fig Interactions at the domain interface. Important interactions at the domain interface in pLGICs of known structure. Top row shows nonconducting conformations, bottom row shows putative conducting conformations or related structures. Interface region of a single subunit is displayed as Cα trace with selected side chains shown as CPK models. (A) Interaction between residues that, upon mutation to alanine, prevent channel activation in ELIC and GLIC. The view is as in Fig 2A. (B) Interaction region between the β1-β2 turn, a conserved arginine at the end of β-10 and the β6-β7 loop. The view is as in Fig 4A. (C). Relationship between the residue at the tip of the β1-β2 turn and a conserved proline at the M2-M3 loop. The view is as in Fig 5A. A–C, figures were prepared with Protein Data Bank entries GLIC pH7 (4NPQ), ELIC (2VL0), GluCl (without agonist, 4TNV), GLYRs (strychnine complex, 3JAD), GLIC (3EHZ), GABA_A_ (4COF), GluClgi (glutamate and ivermectin complex, 3RIF), GlyRgi (glycine and ivermectin complex, 3JAF), and 5-HT_3_ (4PIR).
(TIF)

S4 Fig Electron density of ELIC mutants. (A) Stereo view of the domain interface of the ELIC mutant F116A. 2F_o_–F_c_ electron density (calculated at 3.5 Å and contoured at 1σ, cyan mesh) is shown superimposed on the refined structure. (B) Stereo view of the domain interface of the ELIC mutant Y258A. 2F_o_–F_c_ electron density (calculated at 3.2 Å and contoured at 1σ, cyan mesh) is shown superimposed on the refined structure. (C) Structure of the ELIC mutant T28D. Left, Cα-trace of a subunit with 2F_o_–F_c_ electron density (calculated at 4.5 Å and contoured at 1σ, blue mesh) superimposed. Center, view of the domain interface of the mutant T28D. A stick representation of the model and electron density are shown. Right, Cα-trace of part of the subunit of T28D surrounding the domain interface (grey) is superimposed on WT (orange). (D) Stereo view of the domain interface of the ELIC mutant P254G. 2F_o_–F_c_ electron density (calculated at 3.3 Å and contoured at 1σ, cyan mesh) is shown superimposed on the refined structure. Residues forming a salt bridge that is absent in WT are labeled. A–D, sites of mutation are marked by an asterisk.
(TIF)

S5 Fig ITC. Agonist and antagonist binding to (A) ELIC WT, (B), the ligand-binding site mutant R91A, the double mutants (C), R91A/F116A and (D), R91A/Y258A, and (E), the mutant T28D as determined by ITC. Top graphs show the uncorrected heat exchanged upon addition of the agonist propylamine (left) and the antagonist acetylcholine (right). Bottom graphs show the background (bg) from titrating propylamine (left) or acetylcholine (right) into buffer solution not containing any protein. A fit of the integrated and corrected heat to a binding isotherm is shown in Fig 3F–3I. Experiments were repeated twice with similar results.
(TIF)

S6 Fig Two-electrode voltage clamp electrophysiology. Current response of representative *X*. *laevis* oocytes expressing selected mutants of either ELIC or GLIC at different agonist concentrations. Currents were recorded at −40 mV unless specified otherwise. A bar indicates agonist application. Agonist concentrations (cysteamine in mM for ELIC and pH for GLIC) are shown above. (A) ELIC T28A (recorded at −60 mV), (B), ELIC T28D (recorded at −60 mV), (C), GLIC K32A (recorded at −80 mV), (D), ELIC L29A, (E), ELIC L29 deletion, (F), GLIC P246A, (G), ELIC P254A, (−80 mV), (H), GLIC P246G (−60 mV), (I), ELIC P254G, (−60 mV), (J), comparison of ELIC WT and P254G (both −60 mV). Surface expression of the respective mutants is shown on the right of panels B, E, H and J. Data show averages of at least five different oocytes and are normalized to WT. Background was subtracted. Errors are SEM. (See S1 Data for the raw data used to generate plots shown in panels B, E, H and J).
(TIF)

S7 Fig X-ray structures of GLIC mutants of Pro246. (A) Stereo view of the domain interface of the GLIC mutant P246A. 2F_o_–F_c_ electron density (calculated at 3.3 Å and contoured at 1σ, cyan mesh) is shown superimposed on the refined structure. (B) Cα-trace of residues at the domain interface of P246A (grey) superimposed on GLIC WT (green). (C) Stereo view of the domain interface of the GLIC mutant P246G. 2F_o_–F_c_ electron density (calculated at 3.2 Å and contoured at 1σ, cyan mesh) is shown superimposed on the refined structure. A–C, an asterisk marks the site of mutation. (D) Superposition of Cα traces of the pore forming M2 helices of P246G (grey) and GLIC WT (green). (E) Transmembrane pore of GLIC WT (left) and P246G (right). Helices M2 are shown as sticks, the molecular surface in white. In D and E, the front subunits are omitted for clarity. (F) Region of P246G surrounding His234 (*), which was proposed to play a role in channel activation. Left, structure with 2F_o_–F_c_ electron density superimposed. Right, superposition of the same regions of P246G (grey) and WT (green). A dashed line indicates the interaction between His234 (located on M2) and the backbone of helix M3 in WT.
(TIF)

S8 Fig Kinetic analysis of patch clamp recordings. ELIC WT and mutants were expressed in HEK293 cells. Macroscopic currents recorded from representative excised patches in the outside-out configuration upon fast application and washout of 25 mM propylamine are shown. Application of agonist is indicated by a blue bar. Data was recorded at −50 mV. Time course of current activation and decay was fitted to a single exponential (red traces). Full traces are shown on the left, sections used for fitting in the center and on the right. (A) WT activation and deactivation. Traces in the center and on the right show a fit of the activation and deactivation time constants respectively. (B) WT, desensitization upon prolonged agonist application. Trace on the right shows a fit of the desensitization time constant. (C) P254G, (D), P254G/R255A, (E), R255A, activation and deactivation. Traces in the center and on the right show a fit of the activation and deactivation time constants. (F) R255A, desensitization upon prolonged agonist application. Trace on the right shows a fit of the desensitization time constant. (G) Table displaying the mean and SEM of time constants of 4–8 independent recordings. (See S1 Data for the raw data used to generate this table).
(TIF)

S9 Fig Relationships between pLGICs of known structure. Superposition of subunits of different pLGICs of known structure. Proteins were assigned to one of three distinct groups: (A), GLIC-like conformations, (B), ELIC-like conformations and (C), locally closed or collapsed pore conformations found in a structure of GLIC at pH 7 and in certain mutants of the same channel. A–C, panels show Cα traces of the pore domain and the domain interface from a single subunit of the respective channels. PDB entries are as in S3 Fig. Additional structures shown are GluCll (lipid complex, 4TNW), GlyRg (glycine complex, 3JAE) and GLIC Y250A (4LMK). Structural relationships of helices M2 and M3 within each group are schematically illustrated on the right, the pore axis is indicated by a dashed line. An asterisk in A indicates M2. (D) RMSD of the pore region calculated after least square superposition of equivalent Cα positions in helices M1, M2, and M3 in pentameric structures of different pLGICs. (See S1 Data for the raw data used to generate the plot shown in panel D).
(TIF)

## Abbreviations

Bg: background
EPR: electron paramagnetic resonance
GluCl: glutamate-gated chloride channel
GlyR: glycine receptor
HA-tag: hemagglutinin tag
HEK293: human embryonic kidney 293
HRP: horseradish peroxidase
HRV: human rhinovirus
ITC: isothermal titration calorimetry
MWC: Monod Weyman Changeux
nAChR: nicotinic acetylcholine receptor
non inj.: noninjected
pLGIC: pentameric ligand-gated ion channel
PSI: Paul Scherrer Institute
SEM: standard error of the mean
SD: standard deviation
SLS: Swiss Light Source
WT: wild type
