# Correction: Signal Transduction at the Domain Interface of Prokaryotic Pentameric Ligand-Gated Ion Channels

**DOI:** 10.1371/journal.pbio.1002490

**Published:** 2016-06-07

**Authors:** The PLOS Biology Staff

## Notice of Republication

This article was republished on March 4^th^, 2016 to correct poor figure quality in the PDF version, which was introduced during the typesetting process. The publisher apologizes for these issues with the figure quality. Please download this article again to view the updated version.
