# Supplementary figures and images for: Signal Transduction at the Domain Interface of Prokaryotic Pentameric Ligand-Gated Ion Channels
Source: PLoS Biol. 2016 Mar 4;14(3):e1002393. doi: 10.1371/journal.pbio.1002393 (PMC4778918; doi:10.1371/journal.pbio.1002393)

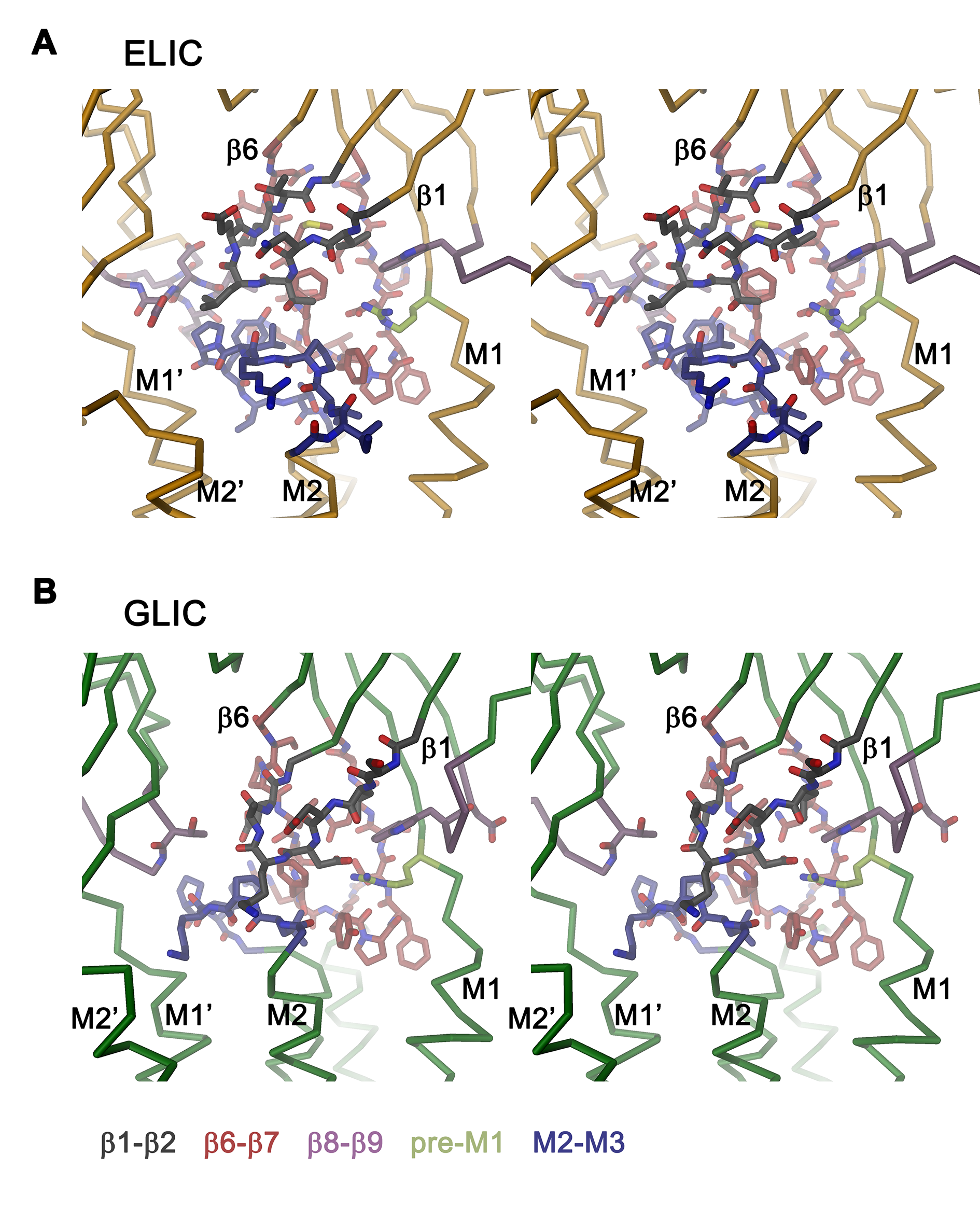

Supplement: S1 Fig — Stereo view of the domain interface of ELIC (A) and GLIC (B). The view is from within the pore parallel to the membrane plane. The respective proteins are displayed as Cα-trace, residues mutated in this study as sticks. Carbon atoms of different regions are shown in unique colors as indicated on the bottom. Selected secondary structure elements are labeled; apostrophes refer to adjacent subunit. (TIF) [file pbio.1002393.s002.tif]

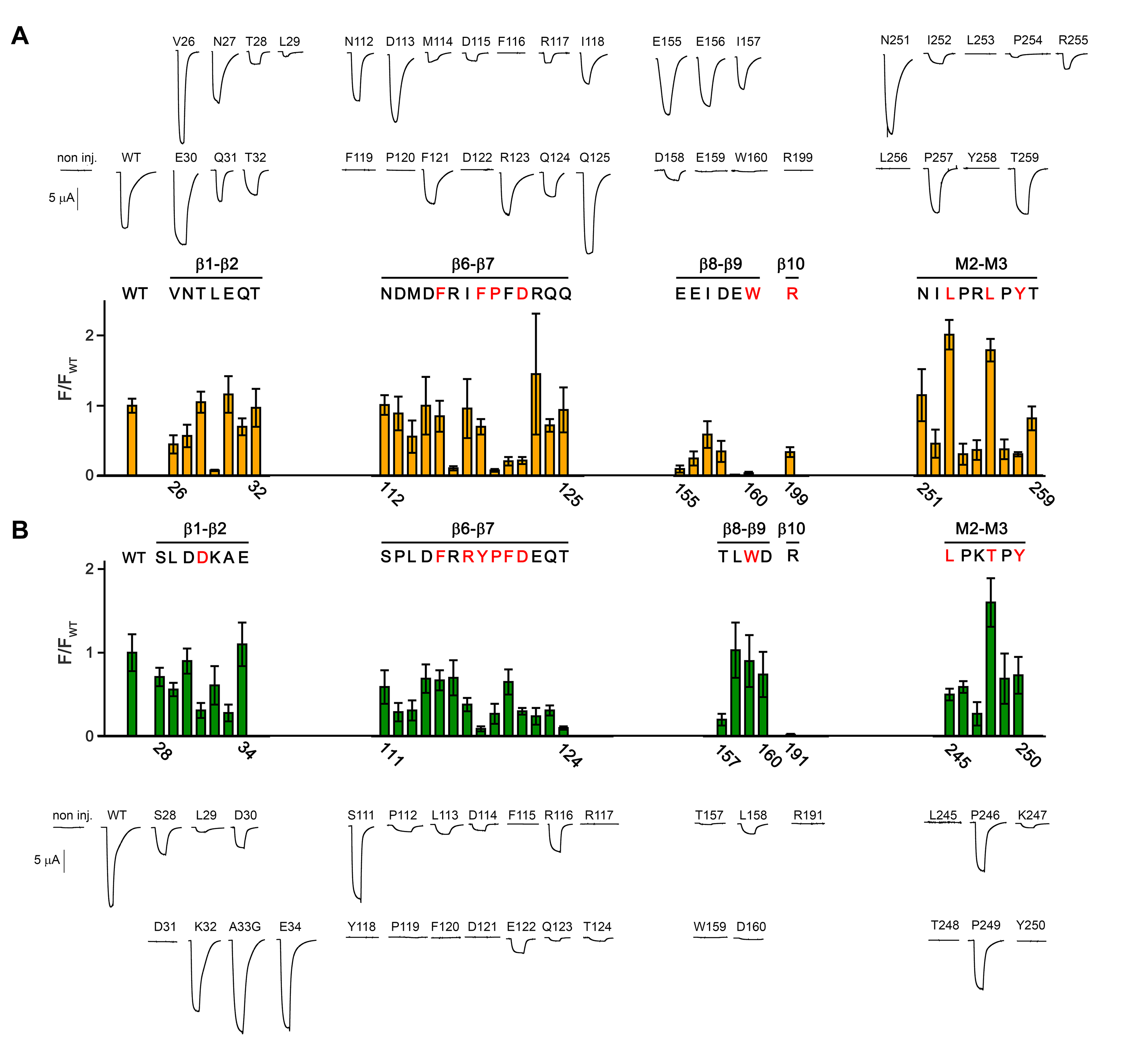

Supplement: S2 Fig — Surface expression levels of alanine mutants of ELIC (A) and GLIC (B) determined by ELISA. Values are averages of 4–6 experiments. Background from empty oocytes was subtracted and signal was normalized to WT. Errors are SEM. The protein sequence is indicated above each chart with nonactivating mutations colored in red. The residue number of selected residues is indicated. Current response at high agonist concentration (ELIC: 25 mM cysteamine, GLIC: pH 4) of representative oocytes expressing the indicated mutants are shown above the respective chart for ELIC and below for GLIC. Currents were either recorded at −40 mV or scaled to the expected value at −40 mV assuming a linear macroscopic conductance. (See S1 Data for the raw data used to generate plots shown in panels A and B). (TIF) [file pbio.1002393.s003.tif]

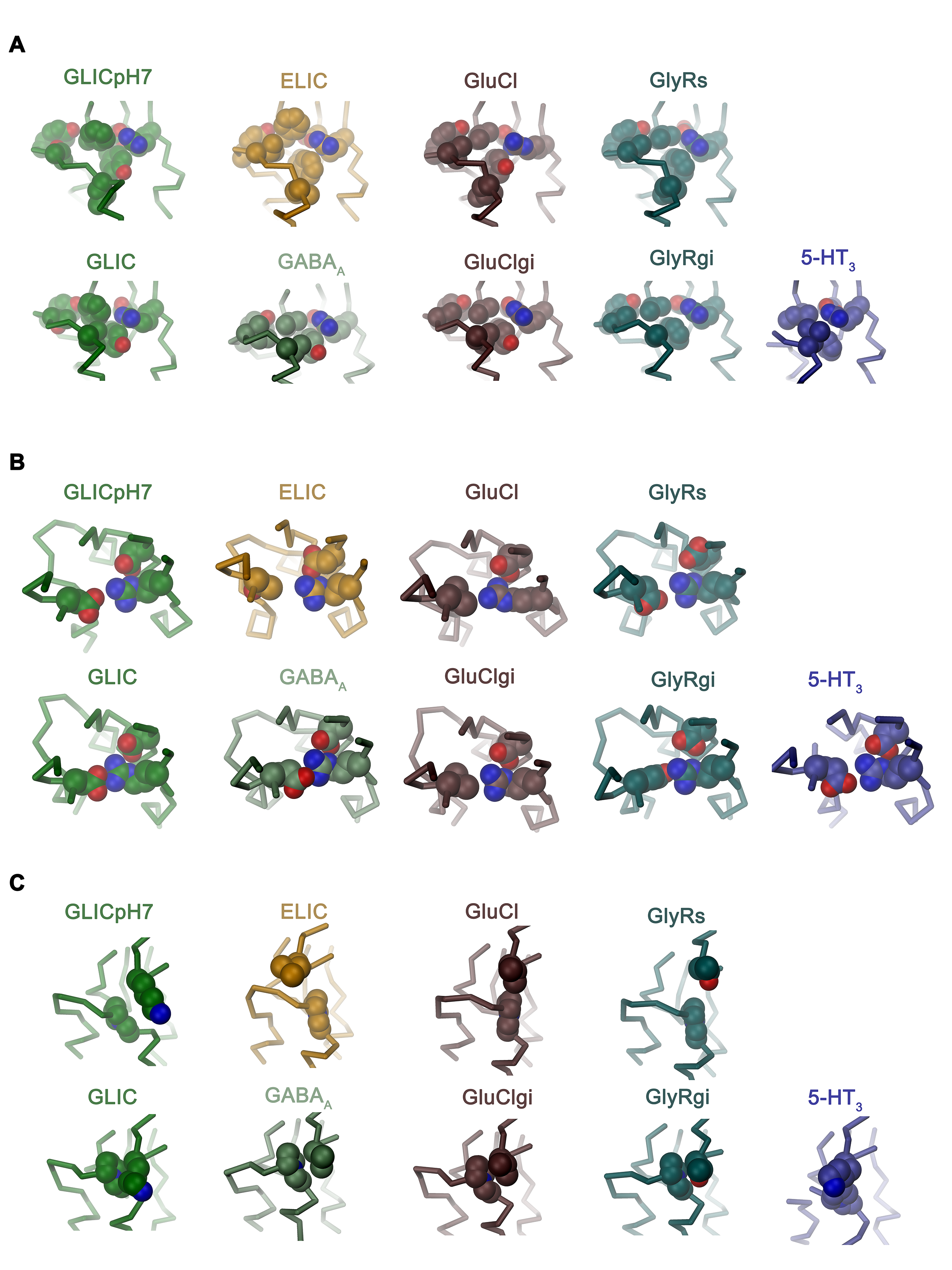

Supplement: S3 Fig — Important interactions at the domain interface in pLGICs of known structure. Top row shows nonconducting conformations, bottom row shows putative conducting conformations or related structures. Interface region of a single subunit is displayed as Cα trace with selected side chains shown as CPK models. (A) Interaction between residues that, upon mutation to alanine, prevent channel activation in ELIC and GLIC. The view is as in Fig 2A. (B) Interaction region between the β1-β2 turn, a conserved arginine at the end of β-10 and the β6-β7 loop. The view is as in Fig 4A. (C). Relationship between the residue at the tip of the β1-β2 turn and a conserved proline at the M2-M3 loop. The view is as in Fig 5A. A–C, figures were prepared with Protein Data Bank entries GLIC pH7 (4NPQ), ELIC (2VL0), GluCl (without agonist, 4TNV), GLYRs (strychnine complex, 3JAD), GLIC (3EHZ), GABAA (4COF), GluClgi (glutamate and ivermectin complex, 3RIF), GlyRgi (glycine and ivermectin complex, 3JAF), and 5-HT3 (4PIR). (TIF) [file pbio.1002393.s004.tif]

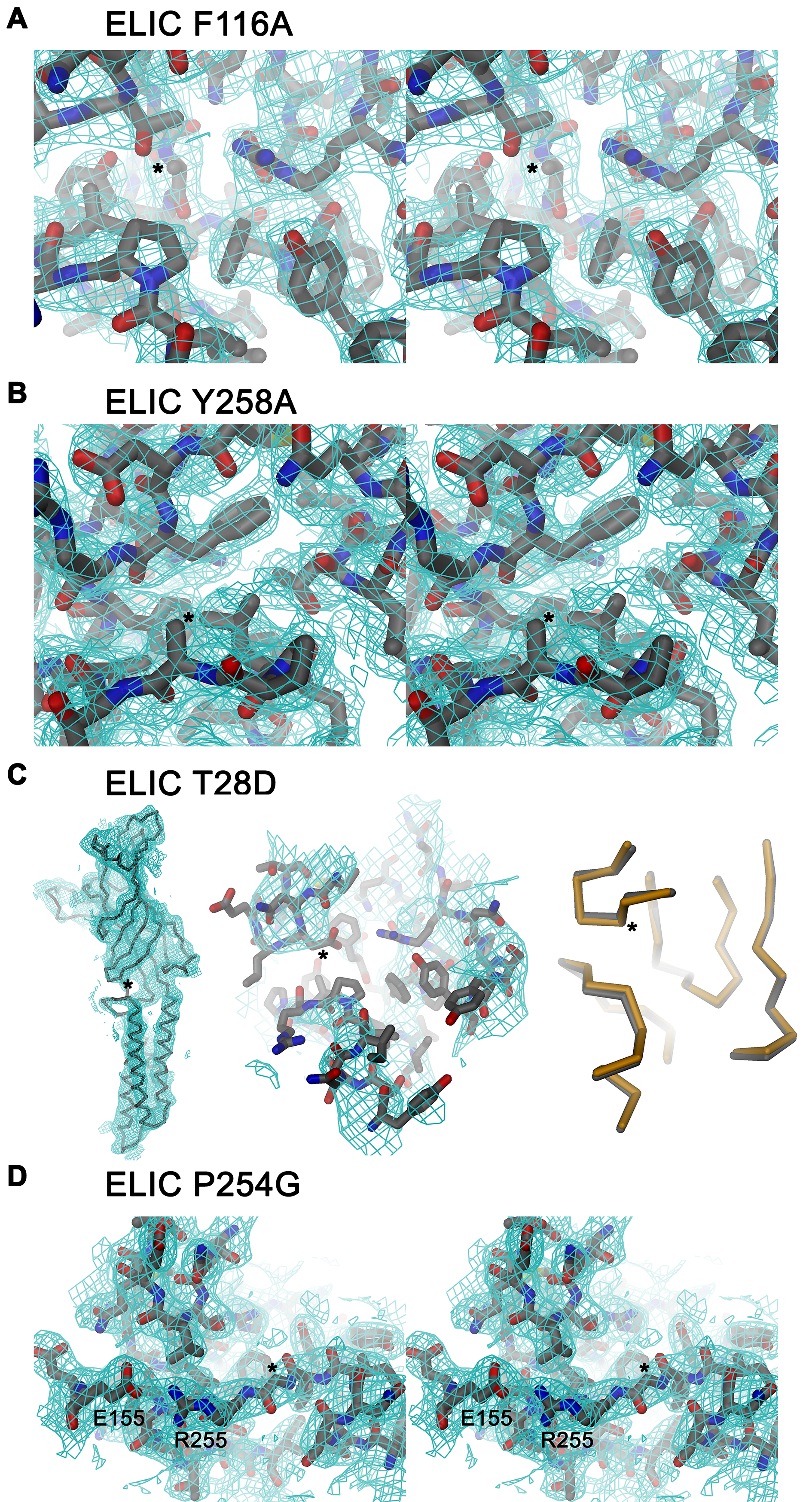

Supplement: S4 Fig — (A) Stereo view of the domain interface of the ELIC mutant F116A. 2Fo–Fc electron density (calculated at 3.5 Å and contoured at 1σ, cyan mesh) is shown superimposed on the refined structure. (B) Stereo view of the domain interface of the ELIC mutant Y258A. 2Fo–Fc electron density (calculated at 3.2 Å and contoured at 1σ, cyan mesh) is shown superimposed on the refined structure. (C) Structure of the ELIC mutant T28D. Left, Cα-trace of a subunit with 2Fo–Fc electron density (calculated at 4.5 Å and contoured at 1σ, blue mesh) superimposed. Center, view of the domain interface of the mutant T28D. A stick representation of the model and electron density are shown. Right, Cα-trace of part of the subunit of T28D surrounding the domain interface (grey) is superimposed on WT (orange). (D) Stereo view of the domain interface of the ELIC mutant P254G. 2Fo–Fc electron density (calculated at 3.3 Å and contoured at 1σ, cyan mesh) is shown superimposed on the refined structure. Residues forming a salt bridge that is absent in WT are labeled. A–D, sites of mutation are marked by an asterisk. (TIF) [file pbio.1002393.s005.tif]

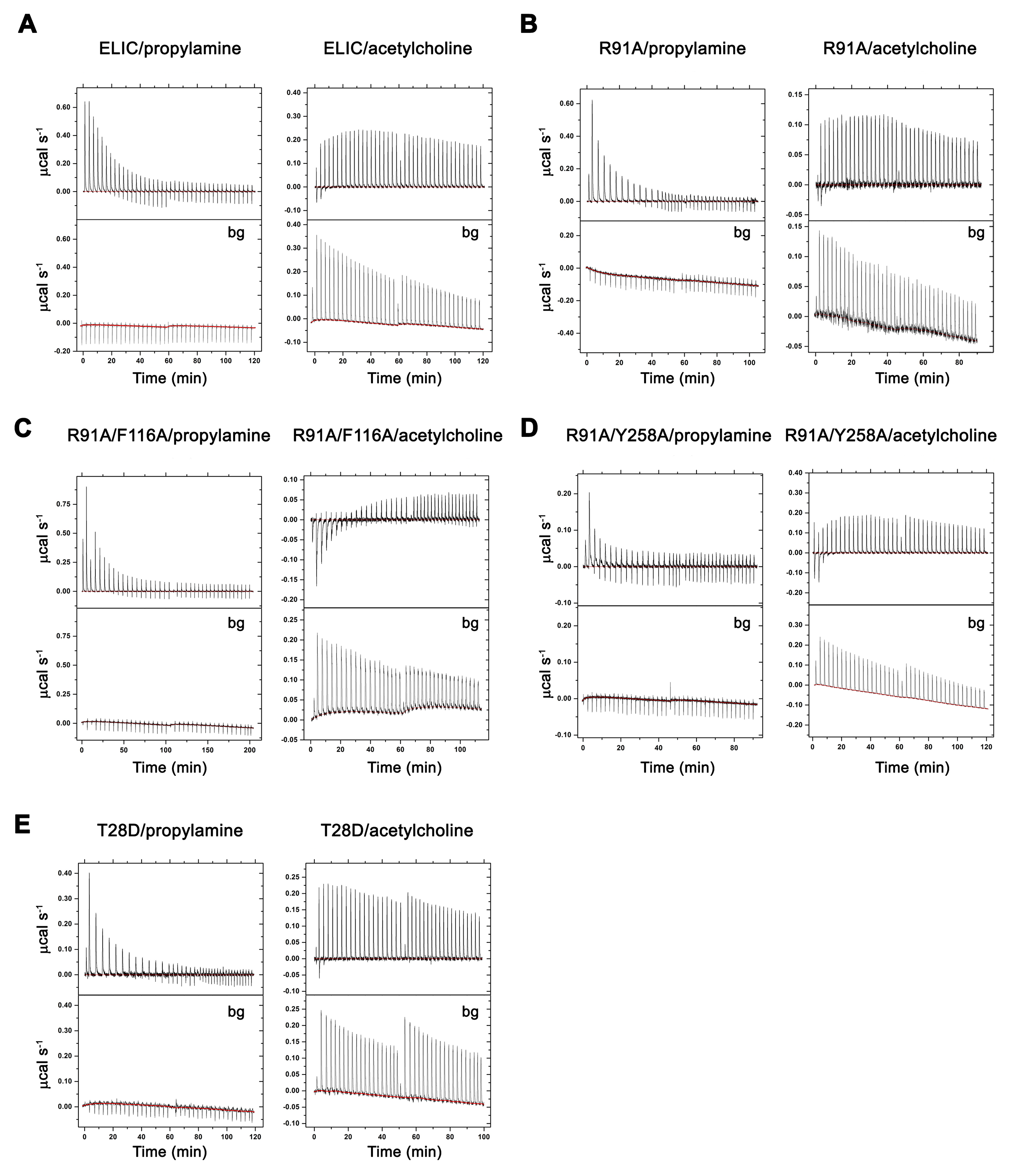

Supplement: S5 Fig — Agonist and antagonist binding to (A) ELIC WT, (B), the ligand-binding site mutant R91A, the double mutants (C), R91A/F116A and (D), R91A/Y258A, and (E), the mutant T28D as determined by ITC. Top graphs show the uncorrected heat exchanged upon addition of the agonist propylamine (left) and the antagonist acetylcholine (right). Bottom graphs show the background (bg) from titrating propylamine (left) or acetylcholine (right) into buffer solution not containing any protein. A fit of the integrated and corrected heat to a binding isotherm is shown in Fig 3F–3I. Experiments were repeated twice with similar results. (TIF) [file pbio.1002393.s006.tif]

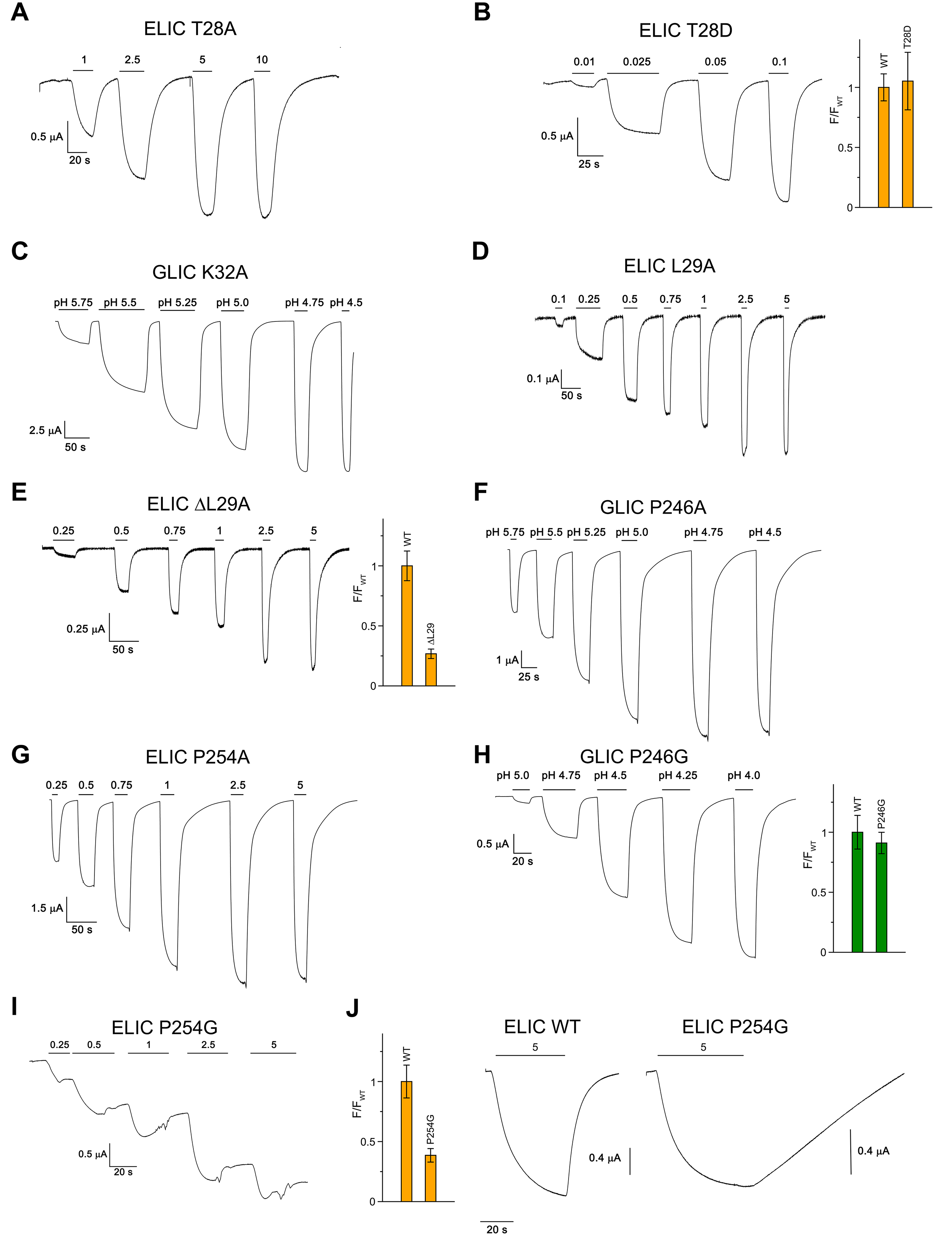

Supplement: S6 Fig — Current response of representative X. laevis oocytes expressing selected mutants of either ELIC or GLIC at different agonist concentrations. Currents were recorded at −40 mV unless specified otherwise. A bar indicates agonist application. Agonist concentrations (cysteamine in mM for ELIC and pH for GLIC) are shown above. (A) ELIC T28A (recorded at −60 mV), (B), ELIC T28D (recorded at −60 mV), (C), GLIC K32A (recorded at −80 mV), (D), ELIC L29A, (E), ELIC L29 deletion, (F), GLIC P246A, (G), ELIC P254A, (−80 mV), (H), GLIC P246G (−60 mV), (I), ELIC P254G, (−60 mV), (J), comparison of ELIC WT and P254G (both −60 mV). Surface expression of the respective mutants is shown on the right of panels B, E, H and J. Data show averages of at least five different oocytes and are normalized to WT. Background was subtracted. Errors are SEM. (See S1 Data for the raw data used to generate plots shown in panels B, E, H and J). (TIF) [file pbio.1002393.s007.tif]

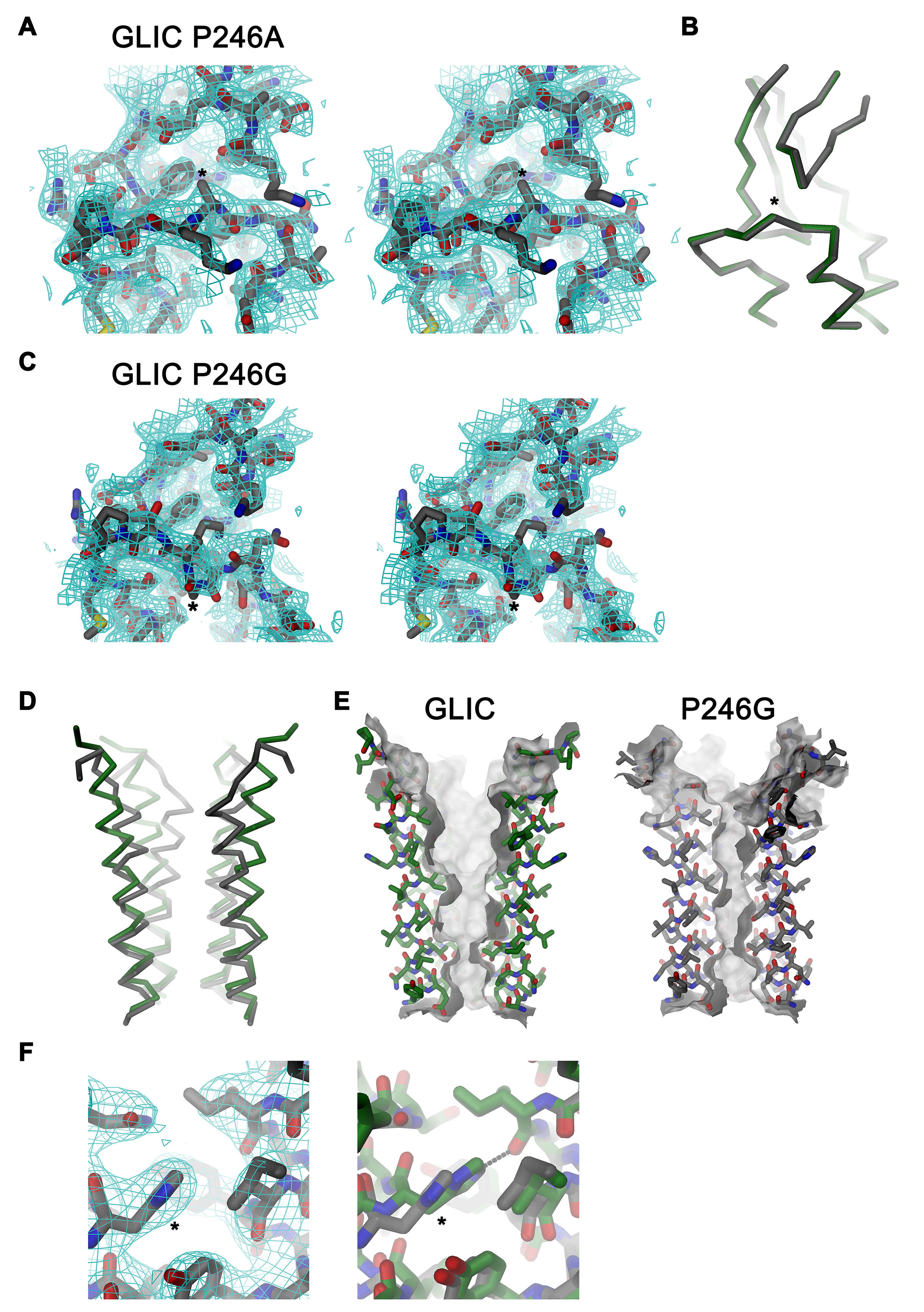

Supplement: S7 Fig — (A) Stereo view of the domain interface of the GLIC mutant P246A. 2Fo–Fc electron density (calculated at 3.3 Å and contoured at 1σ, cyan mesh) is shown superimposed on the refined structure. (B) Cα-trace of residues at the domain interface of P246A (grey) superimposed on GLIC WT (green). (C) Stereo view of the domain interface of the GLIC mutant P246G. 2Fo–Fc electron density (calculated at 3.2 Å and contoured at 1σ, cyan mesh) is shown superimposed on the refined structure. A–C, an asterisk marks the site of mutation. (D) Superposition of Cα traces of the pore forming M2 helices of P246G (grey) and GLIC WT (green). (E) Transmembrane pore of GLIC WT (left) and P246G (right). Helices M2 are shown as sticks, the molecular surface in white. In D and E, the front subunits are omitted for clarity. (F) Region of P246G surrounding His234 (*), which was proposed to play a role in channel activation. Left, structure with 2Fo–Fc electron density superimposed. Right, superposition of the same regions of P246G (grey) and WT (green). A dashed line indicates the interaction between His234 (located on M2) and the backbone of helix M3 in WT. (TIF) [file pbio.1002393.s008.tif]

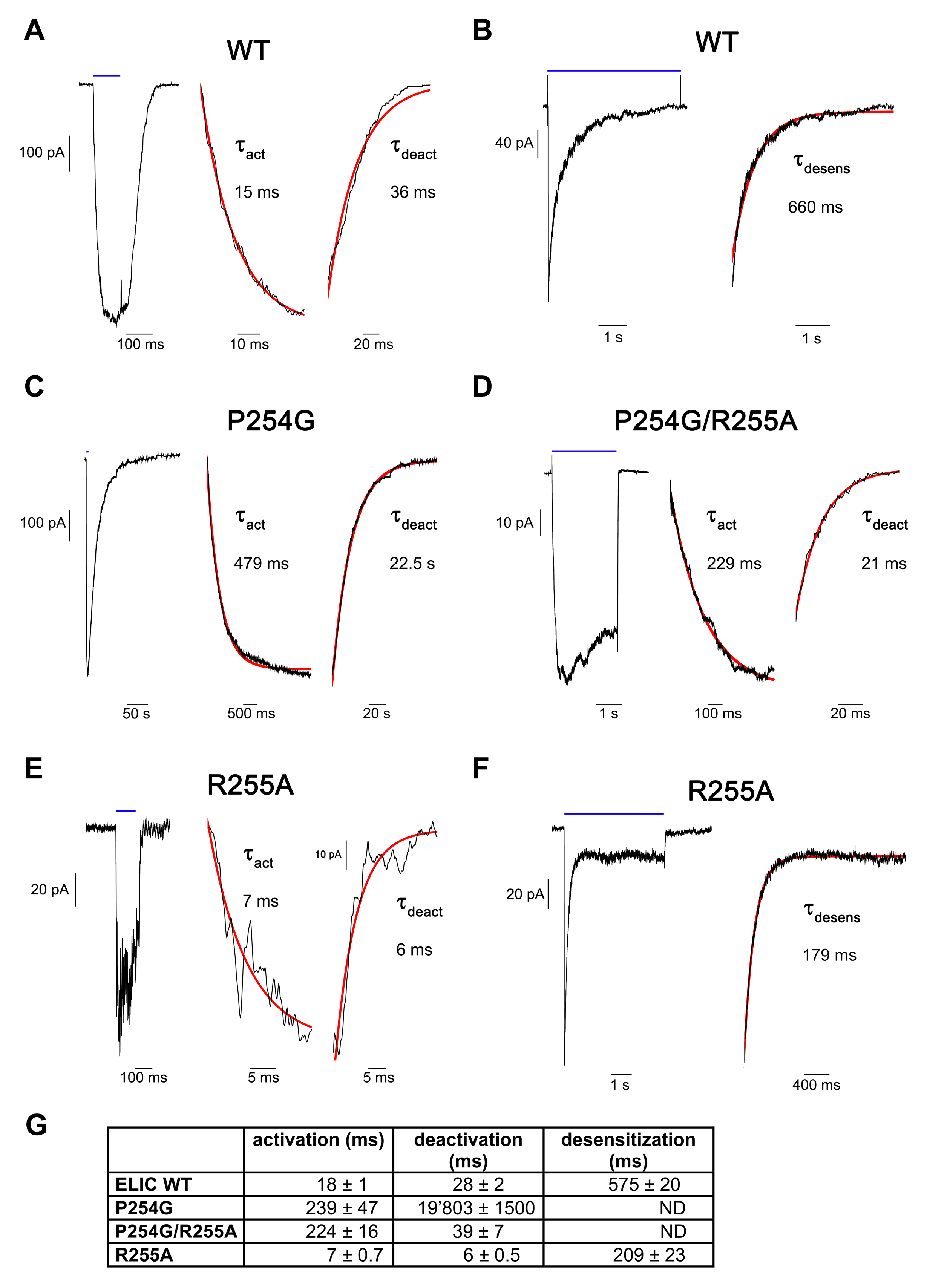

Supplement: S8 Fig — ELIC WT and mutants were expressed in HEK293 cells. Macroscopic currents recorded from representative excised patches in the outside-out configuration upon fast application and washout of 25 mM propylamine are shown. Application of agonist is indicated by a blue bar. Data was recorded at −50 mV. Time course of current activation and decay was fitted to a single exponential (red traces). Full traces are shown on the left, sections used for fitting in the center and on the right. (A) WT activation and deactivation. Traces in the center and on the right show a fit of the activation and deactivation time constants respectively. (B) WT, desensitization upon prolonged agonist application. Trace on the right shows a fit of the desensitization time constant. (C) P254G, (D), P254G/R255A, (E), R255A, activation and deactivation. Traces in the center and on the right show a fit of the activation and deactivation time constants. (F) R255A, desensitization upon prolonged agonist application. Trace on the right shows a fit of the desensitization time constant. (G) Table displaying the mean and SEM of time constants of 4–8 independent recordings. (See S1 Data for the raw data used to generate this table). (TIF) [file pbio.1002393.s009.tif]

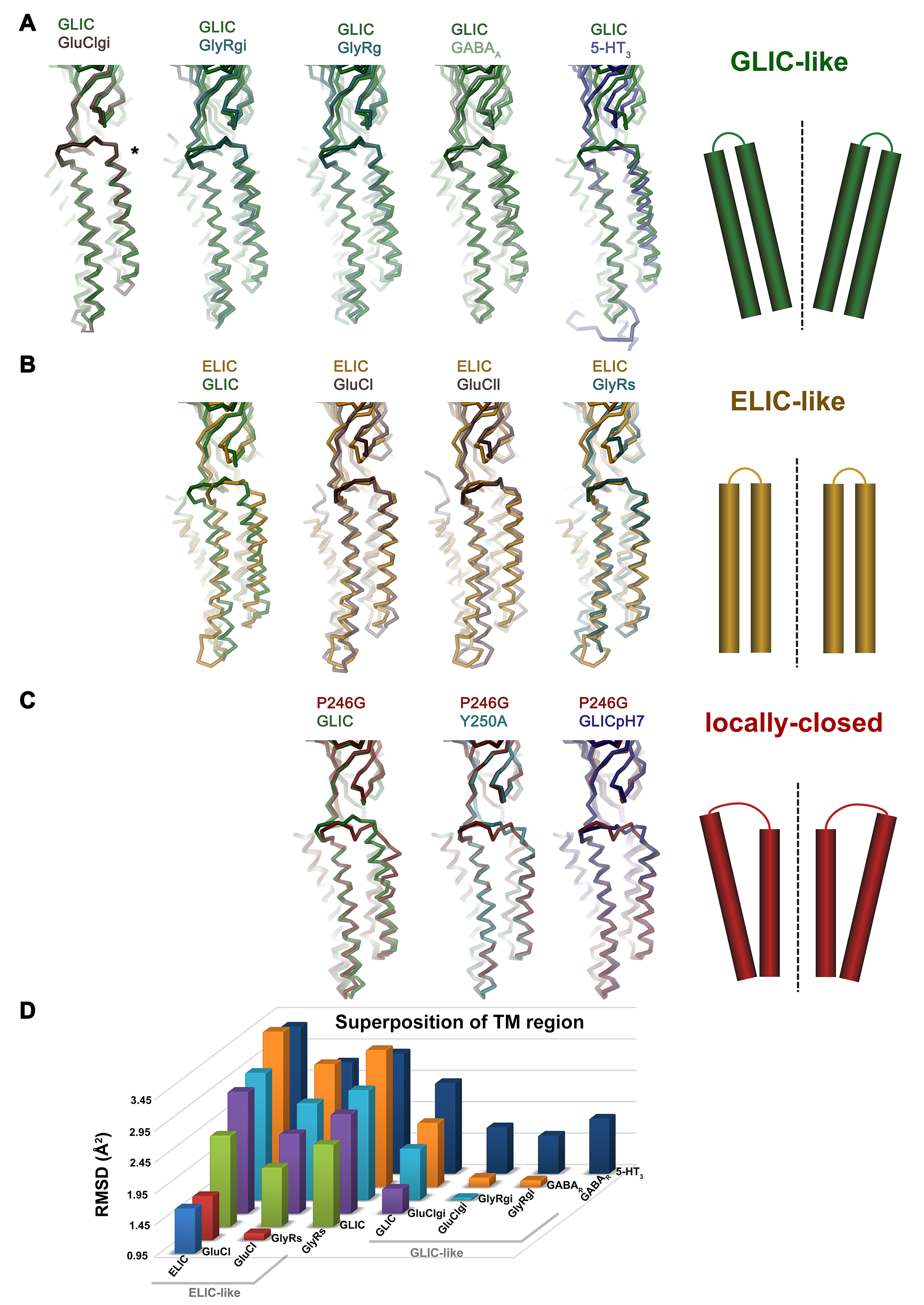

Supplement: S9 Fig — Superposition of subunits of different pLGICs of known structure. Proteins were assigned to one of three distinct groups: (A), GLIC-like conformations, (B), ELIC-like conformations and (C), locally closed or collapsed pore conformations found in a structure of GLIC at pH 7 and in certain mutants of the same channel. A–C, panels show Cα traces of the pore domain and the domain interface from a single subunit of the respective channels. PDB entries are as in S3 Fig. Additional structures shown are GluCll (lipid complex, 4TNW), GlyRg (glycine complex, 3JAE) and GLIC Y250A (4LMK). Structural relationships of helices M2 and M3 within each group are schematically illustrated on the right, the pore axis is indicated by a dashed line. An asterisk in A indicates M2. (D) RMSD of the pore region calculated after least square superposition of equivalent Cα positions in helices M1, M2, and M3 in pentameric structures of different pLGICs. (See S1 Data for the raw data used to generate the plot shown in panel D). (TIF) [file pbio.1002393.s010.tif]
